# Recombination locations and rates in beef cattle assessed from parent-offspring pairs

**DOI:** 10.1186/1297-9686-46-34

**Published:** 2014-05-29

**Authors:** Zi-Qing Weng, Mahdi Saatchi, Robert D Schnabel, Jeremy F Taylor, Dorian J Garrick

**Affiliations:** 1 Department of Animal Science, Iowa State University, Ames, IA 50010, USA; 2 Division of Animal Science, University of Missouri, Columbia, MO 65211, USA

## Abstract

**Background:**

Recombination events tend to occur in hotspots and vary in number among individuals. The presence of recombination influences the accuracy of haplotype phasing and the imputation of missing genotypes. Genes that influence genome-wide recombination rate have been discovered in mammals, yeast, and plants. Our aim was to investigate the influence of recombination on haplotype phasing, locate recombination hotspots, scan the genome for Quantitative Trait Loci (QTL) and identify candidate genes that influence recombination, and quantify the impact of recombination on the accuracy of genotype imputation in beef cattle.

**Methods:**

2775 Angus and 1485 Limousin parent-verified sire/offspring pairs were genotyped with the Illumina BovineSNP50 chip. Haplotype phasing was performed with DAGPHASE and BEAGLE using UMD3.1 assembly SNP (single nucleotide polymorphism) coordinates. Recombination events were detected by comparing the two reconstructed chromosomal haplotypes inherited by each offspring with those of their sires. Expected crossover probabilities were estimated assuming no interference and a binomial distribution for the frequency of crossovers. The BayesB approach for genome-wide association analysis implemented in the GenSel software was used to identify genomic regions harboring QTL with large effects on recombination. BEAGLE was used to impute Angus genotypes from a 7K subset to the 50K chip.

**Results:**

DAGPHASE was superior to BEAGLE in haplotype phasing, which indicates that linkage information from relatives can improve its accuracy. The estimated genetic length of the 29 bovine autosomes was 3097 cM, with a genome-wide recombination distance averaging 1.23 cM/Mb. 427 and 348 windows containing recombination hotspots were detected in Angus and Limousin, respectively, of which 166 were in common. Several significant SNPs and candidate genes, which influence genome-wide recombination were localized in QTL regions detected in the two breeds. High-recombination rates hinder the accuracy of haplotype phasing and genotype imputation.

**Conclusions:**

Small population sizes, inadequate half-sib family sizes, recombination, gene conversion, genotyping errors, and map errors reduce the accuracy of haplotype phasing and genotype imputation. Candidate regions associated with recombination were identified in both breeds. Recombination analysis may improve the accuracy of haplotype phasing and genotype imputation from low- to high-density SNP panels.

## Background

The meiotic exchange of DNA between homologous chromosomes is known as recombination. Recombination events do not take place randomly throughout the genome, but tend to occur in recombination hotspots [1], which are usually small regions in which recombination rate is significantly higher than in surrounding regions. Rates of recombination on different chromosomes are sex-specific, with rates in females being higher near centromeres and rates in males being higher near telomeres [2]. Methodologies for discovering recombination hotspots and descriptions of their properties have been reviewed from the perspectives of mammals [3,4] and plants [5]. Elucidating the characteristics of recombination might help understand the creation and loss of haplotypes and explain genome-wide variation in linkage disequilibrium (LD).

Recombination rates are related to distance from the centromere [5,6], and regional GC content [7,8]. The location and activity of recombination hotspots is regulated both by *cis* and *trans* acting genes [4]. Trans-acting genes, such as *PRDM9* control hotspot activation in mice [9] and humans [1,4], and allow a meiosis-specific protein, SPO11 to initiate recombination [4]. Moreover, *REC8*[10], *RNF212*[10-12] and other loci [11] have been found to influence genome-wide recombination activity in cattle [10] and humans [11,12].

Genome-wide association studies (GWAS) associate genomic variants with a trait of interest to identify positional candidate loci [13,14]. Haplotype-based association tests and imputation from low- to high-density genotyping panels can both improve the power of GWAS to detect QTL [15] and most methods for haplotype phasing can also be used for genotype imputation. Furthermore, the estimation of haplotype phase can use LD information [16] and/or pedigree structure [17]. Statistical models used to infer haplotype phase and impute missing genotypes include Hidden Markov models [18,19], rule-based approaches [20], long-range phasing algorithms [21], and other methods. The importance of haplotype phase estimation and genotype imputation is increasing as large-scale sequencing projects generate genome-wide genotype information.

Effectiveness of genotype phasing and imputation are influenced by marker density, extent of LD, effective population size, marker minor allele frequency (MAF), size of the training population, position on the chromosome, and the extent of pedigree relationships between training and imputed populations [22-24]. Kirk and Cardon [25] pointed out that a small number of genotyping errors can significantly decrease the apparent haplotype frequency and the accuracy of haplotype reconstruction. Haplotype frequencies and counts are also affected by recombination.

Although Sandor et al. [10] have reported estimated heritabilities of recombination rate and the identification of recombination hotspots and quantitative trait loci (QTL) in dairy cattle, recombination rates have been less investigated in cattle than in mice, humans and other mammals. In our study, we quantified recombination rates and their impact on phasing accuracy in two purebred beef cattle populations i.e. Angus and Limousin. Our goals were to: (i) examine the impact of pedigree information, phasing method, and single nucleotide polymorphism (SNP) location errors on the inference of haplotypes, (ii) quantify the impact of recombination on haplotype phasing, (iii) locate recombination hot windows and QTL which influence genome-wide recombination numbers (GRN), and (iv) evaluate the relationship between recombination rate and accuracy of genotype imputation in beef cattle.

## Methods

### Genotype and phenotype

A total of 3570 Angus bulls born between 1955 and 2008, and 2275 Limousin cattle (1319 bulls and 955 daughters) born between 1974 and 2007 that were genotyped with the BovineSNP50 BeadChip (Illumina, San Diego, CA) were used in this study. Genotypes were obtained using DNA samples extracted from semen or hair samples and did not require an approved animal use and care protocol. Genome-wide Mendelian consistency was tested on sire-offspring pairs, and those that failed or had genotype call rates (CR) below 95% were removed. After selection, 2778 Angus and 1485 Limousin parent-verified sire-offspring pairs remained. The average sizes of the 604 Angus and 235 Limousin half-sib families were 4.6 (between 1 and 103) and 6.3 (between 1 and 135), respectively. Individual SNPs with a CR less than 0.95, a MAF less than 0.01, a *p* value for a Hardy Weinberg equilibrium test less than 0.001, or a Mendelian inconsistency rate greater than 0.0024 (95% quantile) were removed. After quality control, 40 990 SNPs across 29 *Bos taurus* (BTA) autosomes in Angus and 38 815 SNPs in Limousin remained, of which 34 788 were in common. Missing 50K genotypes (0.45% and 0.02% of all Angus and Limousin genotypes, respectively) were imputed using BEAGLE3.3 [19]. In order to evaluate the relationship between recombination rate and genotype imputation, a cross-validation study was conducted to quantify the imputation accuracy. Imputation from low- to high-density SNP panels was performed in Angus based on the use of only that subset of 7345 SNPs from the 50K panel that were on the GGP7K panel (GeneSeek, Lincoln, NE) for those animals used in the validation.

### Haplotype phasing

Phasing of haplotypes was performed one chromosome at a time using either the unrelated option in BEAGLE 3.3 [19] or DAGPHASE2.4 [17]. Phasing was first performed using SNP coordinates from the UMD3.1 assembly [26], which is known to contain some errors. The USDA-AIPL linkage map [27] that was constructed from linkage analysis based on the UMD3.1 assembly coordinates was used as an alternative. The comparison of phasing accuracies obtained from these two alternative marker orders was determined for BTA15 as a representative example. The hidden Markov model based on the Viterbi algorithm [28] implemented in BEAGLE was used to reconstruct haplotypes and to impute from low- to high-density SNP panels. In order to increase haplotype phasing accuracy, BEAGLE was set to run 20 iterations of the phasing algorithm and to sample 20 haplotype pairs per individual per iteration. Before using DAGPHASE, the assembly coordinates for the markers were used to generate a genetic map assuming that 1 Mb equals 1 cM. Then, DAGPHASE was used to reconstruct haplotypes based on the output of a directed acyclic graph (DAG) from BEAGLE with scale and shift parameters set at 2.0 and 0.1, respectively.

The number of different haplotypes in every 1 Mb window was counted for each chromosome in the Angus population. Phasing errors can result in erroneous haplotypes that might occur at low frequency, so only haplotypes with a frequency greater than 5% (>5% quantile) in each window were included.

### Estimating recombination events

Recombination events were identified as phase changes in the transmitted gametes by comparing the two reconstructed haplotypes inherited by each offspring with the two reconstructed haplotypes of their sire. Each recombination event was localized to a recombination interval defined by a pair of informative markers for which the phase was known. Haplotype mismatches were not common but were identified when the putative paternally-inherited haplotype of the offspring identified by BEAGLE or DAGPHASE was not identical to either of the haplotypes of the sire. Double crossover events that occurred in intervals less than 2 Mb, animals with more than three crossover events per chromosome, parent-offspring pairs with a haplotype mismatch rate greater than 0.05, crossover events occurring in 1 Mb windows for which the estimated recombination rate was significantly greater than 0.025 or which contained SNPs with a haplotype mismatch rate greater than 0.05 were ignored. Such unlikely crossover events were attributed to either genotyping or phasing errors. The GRN for each parent-offspring pair was calculated as the summation of observed crossover events across the 29 autosomes. On average, one crossover event occurs on a chromosome of size 1 Morgan (M) [29]. Accordingly, the average genome-wide recombination distance per Mb was calculated as the GRN divided by the total length of the 29 bovine autosomes. We found that GRN decreased with increasing family size and that haplotype phasing error rates were inflated in smaller families. As a result, only half-sib families with at least three offspring were retained in the following analysis.

The observed probabilities of 0, 1, 2, 3, >3 crossover events were separately calculated for every autosome. For a given number of crossovers, after removing unlikely crossover events described above, the number of parent-offspring pairs with that number of crossovers was divided by the total number of parent-offspring pairs in the analyzed population, to obtain the observed crossover probability for that chromosome. This produced five observed crossover probabilities for every autosome.

The five expected crossover probabilities were calculated for each autosome based on its length in base pairs assuming that crossover events follow a binomial distribution [30]. Ott [29] pointed out that *N* = 4 would be a reasonable maximum crossover number for chromosomes for which the maximum recombination rate is less than 0.5. The equation to calculate the expected crossover probabilities was:

$$p=\left(\begin{array}{c} N\\ t \end{array}\right){\left(x/N\right)}^{t}{\left(1-x/N\right)}^{N-t},$$

where *p* is the expected crossover probability, *N* is the maximum number of crossover events per chromosome, *t* is the observed number of crossover events (0/1/2/3/4) per chromosome, and *x* is the length (M) of the corresponding chromosome, assuming 100 Mb is 1 M.

The expected genetic length of each chromosome (M) was computed as $\sum_{i=1}^{4}ip_{i}$ where *i* is the number of crossover events (1/2/3/4) on the corresponding chromosome, and *p*_*i*_ is the expected probability of crossover *i*. The expected chromosomal recombination distance per Mb was calculated as the expected genetic length (cM) divided by the physical length (Mb) of the corresponding chromosome.

Recombination rate was estimated for every non-overlapping 1 Mb window to identify recombination hot windows. Some recombination intervals for a particular recombination event could not be localized to positions strictly within a single 1 Mb window. In those cases, a part of the recombination event was considered to have occurred in each window that spanned the recombination interval. The recombination rate in a defined 1 Mb window was computed as:

$$c_{w}=\left(\sum_{k=1}^{n}x_{k}/r_{k}\right)/T,$$

where *c*_*w*_ is the observed window recombination rate, *n* is the total number of recombination events observed on the corresponding chromosome, *x*_*k*_ is the overlap (in Mb) between the 1 Mb window and recombination interval *k*, *r*_*k*_  is the length (in Mb) of the recombination interval, and *T* is the total number of sire-offspring pairs.

### Estimating heritabilities

Genome-wide recombination numbers of sires were treated as phenotypes, thus sires with multiple offspring had repeated records. Narrow sense heritabilities (*h*^*2*^) of GRN were estimated separately for each breed using a repeatability model in ASReml3.0 [31]. The model equation was

y=1μ+Zu+Zp+e,

where **y** is the vector of repeated genome-wide recombination phenotypes for sires, *μ* represents the unknown mean treated as a fixed effect, **u** is the vector of random animal effects with $\mathrm{Var}\left(u\right)=A\sigma_{a}^{2},$ where **A** is the pedigree relationship matrix among sires, **p** is the vector of permanent environmental effects, **1** and **Z** are design matrices, and **e** is the vector of residual effects.

A marker-based heritability was estimated using a BayesC model [32,33] as implemented in GENSEL4.0 software [34]. BayesC assumes that all SNP effects have a common variance, and the prior for that variance has a scaled inverse Chi-square distribution. The model equation was:

$$y_{i}=\mu +\sum_{j=1}^{k}z_{\mathrm{ij}}s_{j}+e_{i},$$

where *y*_*i*_ is the average GRN for sire *i*, *μ* is the population mean, *k* is the number of SNP, *z*_*ij*_ is genotype code (0/1/2) for SNP *j* in sire *i*, *s*_*j*_ is the random effect for SNP *j* with $\left\{\begin{array}{c} s_{j}\sim N\left(0,\sigma_{s}^{2}\right),\mathrm{with}\;\mathrm{probability}\;1-\pi \\ s_{j}=0,\mathrm{with}\;\mathrm{probability}\;\pi \end{array}\right)$, and *e*_*i*_ is a weighted residual effect. Parameter *π*  was set to 0 in this study. BayesC with *π*  equal to 0 is equivalent to GBLUP (Genomic Best Linear Unbiased Predictor), except that the variance components are treated as unknown with scaled inverse chi-squared priors. Markov chain Monte Carlo (MCMC) sampling with 41 000 iterations in which the first 1000 samples were discarded for burn-in, was used to make inferences about variance components and heritability. The weighting factor (*w*_*n*_) [35] for residual variance was calculated as:

$$w_{n}=\frac{1-h^{2}}{ch^{2}+\frac{1+\left(n-1\right)t}{n}-h^{2}},$$

where *h*^2^ is the narrow sense heritability estimated from pedigree, *c* is the proportion of genetic variation that could not be explained by markers, *t* is the repeatability, and *n* is the number of observations for the sire. In this study, *c* was assumed to be equal to 0.40 for both Angus and Limousin, according to [36].

### Genome-wide association study

Mapping QTL that influence sire mean GRN was undertaken using the BayesB method [37] with weighting factors defined as for the above model implemented in GENSEL4.0 software [34]. BayesB assumes that each SNP effect is drawn from a distribution with a locus-specific variance with scaled inverse Chi-square prior distributions, and that a fraction (1 - *π*) of the markers have non-zero effects. Parameter *π*  was assumed to be equal to 0.995, which results in about 0.5% of the SNPs fit in the model at each iteration. Based on simulations, Sun et al. [38] showed that the BayesB method could precisely map QTL. The genome was divided into non-overlapping 1 Mb windows and the posterior distribution of the percentage of genetic variance attributed to each window was constructed from the MCMC samples (e.g. [39]). The expected percentage of genetic variance explained by each of the ~2600 1 Mb windows is about 0.04% under a polygenic model. Windows that explained at least 0.2% (5 fold the expected percentage) of the genetic variance [39], and extended regions on either side of these windows (±2 Mb) were considered to represent QTL. Unpublished simulations using beef cattle genotypes showed that the location of a QTL can be up to 2 Mb up- or downstream of a 1 Mb window that explains a high proportion of genetic variance. The window posterior probabilities of association (WPPA) of candidate windows (i.e. with at least 0.2% genetic variance) was at least 1.5-fold greater than the average WPPA of 1 Mb windows across the genome. WPPA is the posterior probability that a window harbors a QTL, which is the proportion of samples for which at least one SNP in the window was included with a non-zero effect. The SNP that had the highest posterior probability of inclusion (PPI) and explained the largest proportion of genetic variance in each central candidate window was identified as a candidate SNP. The PPI is estimated as the percentage of MCMC samples in which a given SNP had a non-zero effect. The proportion of genetic variance explained by each candidate SNP was assessed as the difference in genetic variance explained by the window when it included or excluded the candidate SNP. Significance of the effect of the candidate SNP was evaluated in an animal model with ASReml3.0 [31], by fitting the SNP genotype as a fixed class effect. Bonferroni adjustment was applied to *p* values from that single SNP analysis by accounting for the number of effective chromosome segments across the genome (*M*_*e*_), which was calculated as [40]:

$$M_{e}=2N_{e}\mathrm{Lk}/\log\left(N_{e}L\right),$$

where  *N*_*e*_ is the effective population size, *L* is the average length of a chromosome in Morgan (~1 M), and *k* is the number of chromosomes (*k* = 30). In this study,  *N*_*e*_  was assumed to be equal to 545 for Angus and 91 for Limousin, based on [41].

Using the human-bovine comparative map implemented in VCMap3.0 [42], orthologous human genome regions corresponding to candidate bovine windows were located. Positional candidate genes within these orthologous human regions were identified using the NCBI Human Genome Overview Build 36.3 (http://www.ncbi.nlm.nih.gov/mapview/). A list of previously published human candidate genes related to meiosis, recombination, or the cell cycle were extracted from OMIM [43]. Using VCMap3.0 [42] or Ensembl (http://www.ensembl.org), locations of the bovine orthologs of these genes were mapped to the bovine genome. These locations were used to test for concordance between locations of candidate genes and identified QTL.

### Imputation from low- to high-density and cross-validation of imputation accuracy

Cross-validation was used to determine the accuracy of BEAGLE imputation from 7K to 50K SNPs in the Angus dataset. The genotyped bulls were clustered into five groups using a K-means clustering method based on additive genetic relationships between animals [44]. The aim of this method was to increase within-group and decrease between-group relationships. Four testing groups were used for phasing haplotypes from the 50K SNP genotypes, while imputation from the 7K SNP panel was performed in the fifth validation group. This was repeated with each of the five groups being treated once as the validation group. The 7K SNP genotypes were extracted from the 50K SNP genotypes for the validation group.

Accuracies of imputation were quantified per marker and summarized per chromosome and per animal. The imputation accuracy was evaluated as the fraction of the imputed genotypes that were identical to the original genotypes on the 50K SNP panel in the validation group. Imputation accuracy was also quantified separately in every 1 Mb window along each chromosome.

Levels of LD between every two adjacent SNPs were evaluated as *r*^2^, the squared simple correlation between genotypes of two adjacent SNPs using R software [45]. These measures of LD between adjacent markers were averaged to provide a single measure of LD for each 1 Mb window.

## Results

### Phasing accuracy and crossover probability

**Figure 1:**
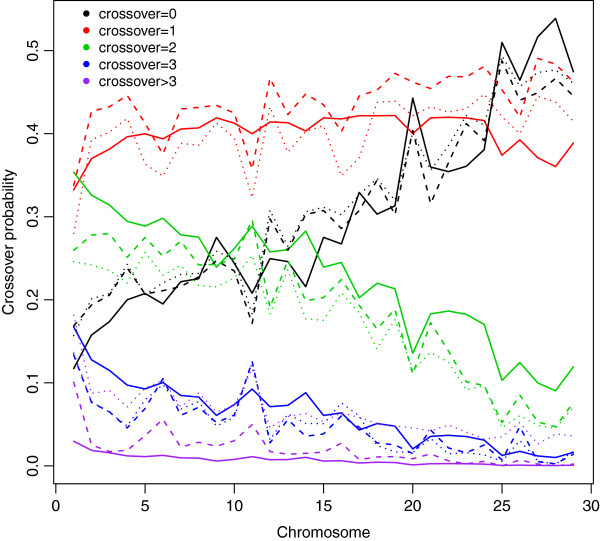
**Probability of zero (black), one (red), two (green), three (blue), and more than three (purple) crossover events per chromosome for each of the 29 bovine autosomes in Angus.** Solid line represents expected probability calculated by Karlin’s map function, dashed line represents observed probability using DAGPHASE, and dotted line represents observed probability using BEAGLE.

Figure 1 shows that the probability of zero crossover events per chromosome increased with decreasing chromosome length, while the probability of two or more crossover events decreased. The observed proportion of individuals with more than three crossover events was higher than the expected value for all autosomes. In general, DAGPHASE, which uses linkage information from parent-offspring relationships, produced a distribution of crossovers for which the observed frequencies were closer to the expected values, indicating that it was superior for phasing compared to BEAGLE with pedigree ignored.

**Figure 2:**
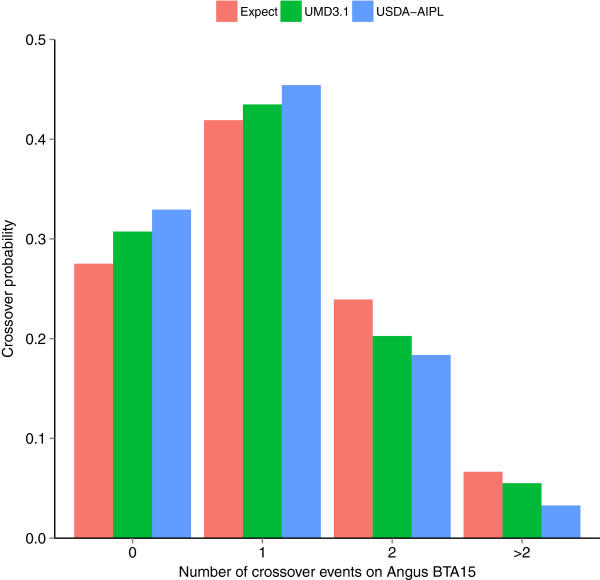
Representative example of expected and observed crossover probabilities on autosome 15 in Angus using UMD3.1 (green) and USDA-AIPL coordinates (blue).

The distributions of observed numbers of crossovers on BTA15 were close to the expected values for both the Angus and Limousin breeds, except for the proportion of more than two crossover events per meiosis [see Additional file 1: Figure S1A], which exceeded the expected values. Possible reasons for the phasing errors that likely caused the discrepancies between expected and observed crossover probabilities are small half-sib family sizes (median size was 2 in both populations), limited numbers of parent-offspring pairs, and errors in some mapped SNP locations. Both the Angus (41.4%) and Limousin (36.6%) breeds had a large proportion of half-sib families represented by only one son. Compared to Angus, the Limousin breed had a higher probability of more than two crossover events per meiosis, probably because of its smaller sample size which reduces the accuracy of haplotype phasing. The higher accuracy in Angus compared to Limousin was also observed for autosomes other than BTA15 [see Additional file 1: Figure S1B]. It has been found that the larger the phasing sample size, the greater the haplotype phasing accuracy [15].Figure 2 compares observed probabilities of crossover events on Angus BTA15 using UMD3.1 versus USDA-AIPL locus coordinates. A total of 1304 SNPs were assigned based on the UMD3.1 and 1262 SNPs based on the USDA-AIPL, with 1234 common SNPs. The estimated probability of more than two crossover events using USDA-AIPL coordinates was smaller than that using the UMD3.1 coordinates, which suggests that a better genome assembly can improve the accuracy of phasing.

### Number of haplotypes and recombination rates

**Figure 3:**
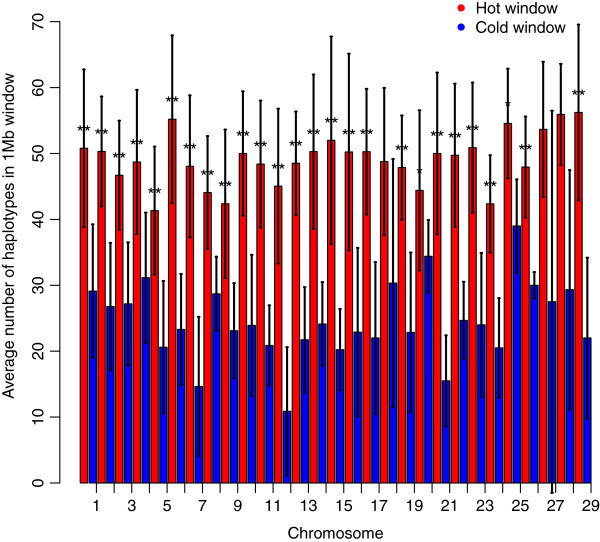
**Relationship between number of haplotypes and crossover rate within 1 Mb windows across the 29 autosomes in Angus.** Number of haplotypes in hot (red) and cold windows (blue), which were defined as windows with a recombination rate ≥ 0.02 (≥1.5 standard deviations from the mean) and windows with recombination rate ≤ 0.004, respectively; *indicates a significant difference with *p* < 0.05, and **indicates a significant difference with *p* < 0.01 for a paired t-test.

The average number of unique haplotypes per 1 Mb windows (±SD) was 37.1±13.8 in Angus. Despite the presence of outliers, there was a linear relationship between number of haplotypes and recombination rate [see Additional file 2: Figure S2]. The number of haplotypes declined with decreasing window-wide recombination rates, because new haplotypes are formed by recombination. Recombination hot or cold windows were defined as windows with recombination rates greater than 0.02 (≥1.5 standard deviations from the mean) and lower than 0.004, respectively. These definitions differ from those previously used for dairy cattle (60 Kb window) [10] and humans (<2 Kb window) [4], because of the different lengths of the defined window. The average number of SNPs (±SD) was 17.8 (±4.5) in hot windows and 13.7 (±5.8) in cold windows. The average numbers of haplotypes (±SD) in hot and cold windows were adjusted for the corresponding average number of SNPs. The number of haplotypes was equal to 50.9±13.3 (ranging from 23.4 to 99.5) in hot windows, and 24.5±6.8 (ranging from 7.4 to 47.2) in cold windows, respectively (Figure 3). The correlation coefficient between the average number of unique haplotypes within each window and the recombination rate in that 1 Mb window was 0.64. All autosomes showed significant disparities in numbers of haplotypes per 1 Mb windows between hot and cold windows.

### Analysis of genome-wide recombination number (GRN)

**Figure 4:**
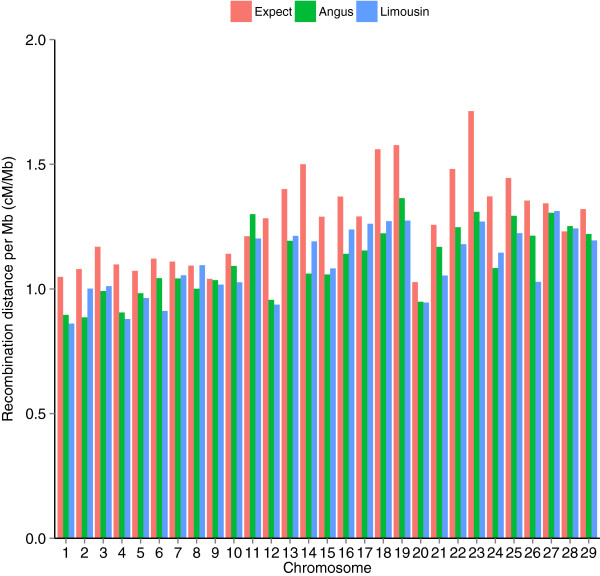
Expected and estimated recombination distance per Mb (cM/Mb) for the 29 bovine autosomes in Angus (green) and Limousin (blue).

A total of 76 186 and 32 052 informative crossover events were identified in Angus and Limousin gametes, respectively. The physical length of the 29 bovine autosomes is 2511.4 Mb (UMD3.1), which corresponds to an average genetic length of 3097 cM (Table 1). On average, the genome-wide recombination distance per Mb across the 29 autosomes was 1.23 cM/Mb. BTA20 had the lowest, and BTA23 the highest cM per Mb ratio. Figure 4 shows the expected and estimated recombination distances per Mb for the 29 bovine autosomes. Recombination distances per Mb differed between chromosomes; short chromosomes had greater genetic distances per Mb than long chromosomes [2]. Chromosomal recombination distances per Mb estimated in the two breeds were similar, with a correlation coefficient of 0.84 between Angus and Limousin. However, the recombination distances per Mb were lower than the expected values for most autosomes, which suggests that conservative filtering of unlikely crossover events leads to an underestimation of the chromosome-specific recombination distance per Mb.

**Table 1:** Physical length, estimated genetic length and recombination distance per Mb of bovine autosomes

**Chromosome**	**Genetic length (cM)**^**a**^	**Physical length (Mb)**^**b**^	**cM/Mb**
1	166.0	158.3	1.05
2	148.0	137.1	1.08
3	141.8	121.4	1.17
4	132.5	120.2	1.10
5	130.0	121.2	1.07
6	134.2	119.5	1.12
7	125.5	112.6	1.11
8	124.4	113.4	1.10
9	110.3	105.7	1.04
10	118.9	104.3	1.14
11	129.9	107.3	1.21
12	117.3	91.2	1.29
13	118.3	84.2	1.40
14	127.4	84.7	1.51
15	110.3	85.3	1.29
16	112.4	81.7	1.38
17	97.0	75.2	1.29
18	103.2	66.0	1.56
19	100.8	64.1	1.57
20	73.7	72.0	1.02
21	90.2	71.6	1.26
22	91.4	61.4	1.49
23	90.0	52.5	1.71
24	85.8	62.7	1.37
25	62.0	42.9	1.45
26	69.8	51.7	1.35
27	60.9	45.4	1.34
28	57.3	46.3	1.24
29	68.0	51.5	1.32
Total	3097.3	2511.4	1.23

^a^Estimated using Karlin’s map function; ^b^bovine UMD3.1 genome assembly.

Taking BTA15 as an example, the correlation between window recombination rates in the Angus and Limousin breeds was equal to 0.56, and recombination rates in a 1 Mb window varied from 0 to over 0.02 [see Additional file 3: Figure S3A]. A large number of recombination hot and cold windows were detected across the chromosome. Since bovine chromosomes are acrocentric, with the centromere at the proximal chromosome end, recombination rates were relatively low in that region. Reduced information at the proximal end of the chromosome could also lead to a low accuracy of detected recombination events. As shown in Additional file 3: Figure S3B, the location of hot and cold windows for recombination was consistent for the two breeds across the genome, although, in some instances, window shifts existed, such that a higher recombination rate for Angus corresponds to a lower recombination rate for Limousin and vice versa. Across the genome, the correlation of 1 Mb window recombination rate between the Angus and Limousin breeds was high, with a correlation coefficient of 0.49. The average window recombination rates per 1 Mb (±SD) were equal to 0.0099±0.0052 and 0.0088±0.0053 in Angus and Limousin breeds, respectively. A total of 427 and 348 hot windows were identified in Angus and Limousin, respectively, of which 166 were in common. Hot windows were found in both the proximal and distal chromosome ends, while cold windows clustered around the middle of each chromosome and the proximal chromosome end.

The average number of recombination events per chromosome differed between autosomes. Longer autosomes tended to have more recombination events. The average GRN (±SD) was equal to 27.4±5.0 in Angus and 26.9±4.8 in Limousin. These values were close to the paternal recombination numbers of 27.6 reported by Chowdhury et al. [11] and 27.0 reported by Kong et al. [21] in humans. GRN did not differ significantly between the breeds [see Additional file 4: Figure S4]. Estimates of GRN slightly decreased with increasing family size (with a correlation coefficient near -0.1), as did the observed variation of GRN across families, which is probably due to an increase of phasing errors in small families [see Additional file 5: Figure S5].

### Estimated heritability and QTL for genome-wide recombination number

The pedigree-based estimates of heritability of GRN (±SE) by ASReml3.0 [31] were equal to 0.26±0.030 and 0.23±0.042 and estimates of repeatability were equal to 0.33±0.027 and 0.30±0.038 in Angus and Limousin, respectively. However, estimates of marker-based heritability of GRN (±SE) by BayesC in GENSEL4.0 software [34] were slightly lower, i.e. 0.17±0.039 in Angus and 0.14±0.031 in Limousin. Results reported in Saatchi et al. [44] demonstrate that the marker-based heritability of routinely recorded traits (e.g. calving ease) of American Angus beef cattle was sometimes lower than the value of the pedigree-based heritability. This suggests that markers only captured a proportion of the genetic variance estimated from pedigree.

**Figure 5:**
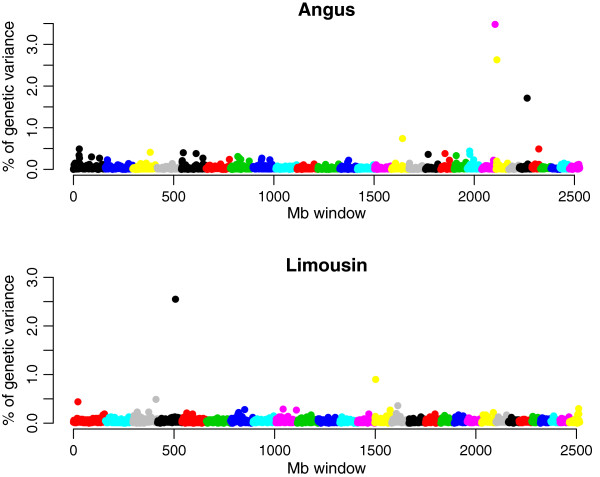
Proportion of genetic variance explained by 1 Mb windows across the genome for genome-wide recombination number (GRN) in Angus and Limousin.

Manhattan plots of the proportion of genetic variance explained by each 1 Mb window across the genome for GRN in Angus and Limousin are in Figure 5. The number of windows explaining at least 0.2% of the additive genetic variance was 35 in Angus and 22 in Limousin. The cumulative variance explained by those windows was equal to 17.8% in Angus and 8.2% in Limousin. Windows that exceeded 0.2% additive genetic variance and had 1.5-fold average WPPA were considered to be significant for further study [see Additional file 6: Table S1]. Different candidate SNPs were identified within each window in Angus and Limousin. The highest proportion of genetic variance (3.48%) was explained by a 1 Mb window located at 67 Mb on BTA21 for Angus, which had a high WPPA (0.45) and a significant SNP accounting for 3.42% of the genetic variance. The most significant region in the Limousin breed was a 1 Mb window located at 89 Mb on BTA4 and explained 2.55% of genetic variance. Positional candidate genes [see Additional file 6: Table S1], that have been reported to be involved in meiotic recombination, DNA replication, DNA repair or the cell cycle [43] were detected within or near (±2 Mb) significant windows but only in Angus; *RAD51C*, *RAD52C*, and *XRCC3* are involved in both meiotic recombination and repair of damaged DNA, while *PRMT8* is only involved in DNA repair, whereas *PTPRM* and *RAD17* regulate cellular processes, such as differentiation and cell cycle checkpoint control.

### Imputation accuracy and recombination rate

In Angus, the average imputation accuracy across animals was equal to 0.981, ranging from 0.81 to 1.00, and the average imputation accuracy per chromosome was also equal to 0.981, ranging from 0.97 to 0.99 [see Additional file 7: Table S2]. BTA21 had the lowest imputation accuracy (0.973), while BTA4 had the highest accuracy (0.985). The average marker density (i.e. average distance in kb between two adjacent markers) was equal to 61.0 kb, ranging from 54.6 to 70.9 kb, and the average *r*^*2*^ between adjacent markers within each 1 Mb window was 0.237, ranging from 0.192 to 0.269. Imputation accuracy increased slightly as marker density and *r*^*2*^ increased.

In Additional file 8: Figure S6A, Angus bulls were grouped according to the number of observed crossover events per chromosome. The average imputation accuracy (±SD) in groups with no, one, two and more than two crossover events was equal to 0.986±0.00835, 0.983±0.0191, 0.981±0.0203, and 0.980±0.0215, respectively. Imputation accuracy decreased only slightly as the number of crossover events increased. Taking BTA1 as an example, imputation accuracy was highest in individuals with no crossover events, and lowest in individuals with more than two observed crossover events due to a higher risk of phasing errors.

**Figure 6:**
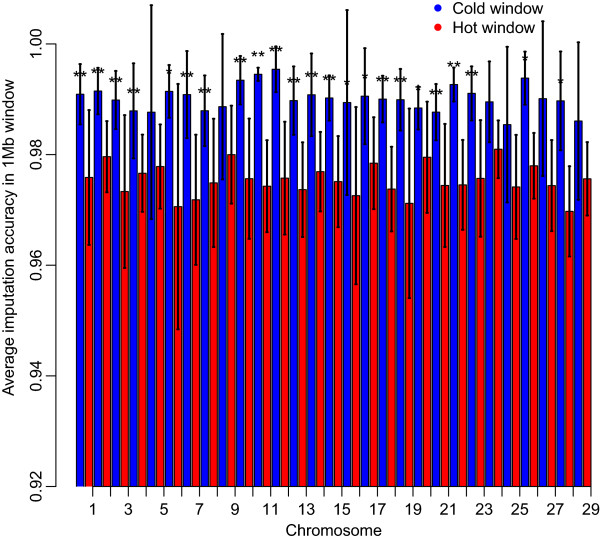
**Imputation accuracy in recombination hot (red) and cold windows (blue).** Hot (red) and cold windows (blue), which were defined as windows with a recombination rate ≥ 0.02 (≥1.5 standard deviations from the mean) and windows with recombination rate ≤ 0.004, respectively; *indicates a significant difference with *p* < 0.05, and **indicates a significant difference with *p* < 0.01 for a paired t-test.

Window-wide imputation accuracy decreased with increasing recombination rate [see Additional file 8: Figure S6B]. The correlation coefficient between window-wide imputation accuracy and recombination rate was equal to -0.49 and the regression coefficient of imputation accuracy on recombination rate was equal to -1.0. Average imputation accuracies of 0.975 (ranging from 0.913 to 0.995), and 0.990 (ranging from 0.927 to 1.00) were found in hot and cold windows, respectively. Figure 6 shows that imputation accuracy was lower in recombination hot windows than in cold windows.

## Discussion

### Impact of phasing methods

Accuracy of phasing haplotypes was quantified in relation to recombination events. DAGPHASE [17], which uses linkage information from parent-offspring relationships was superior to using BEAGLE [19] with relationships for phasing haplotypes ignored. DAGPHASE extracts both population LD and linkage information for phasing, rather than relying on LD alone [17]. To infer haplotypes of offspring with both genotyped parents, parental haplotypes and linkage information were used. For offspring with one genotyped parent, linkage and LD information were jointly used by DAGPHASE, while only LD information extracted from the BEAGLE-produced DAG file was used to determine haplotypes of individuals with both parents non-genotyped. DAGPHASE uses a diploid Hidden Markov model (HMM) and Viterbi algorithm with linkage and LD information to improve phase reconstruction [17]. BEAGLE also assumes a HMM but uses EM-style updating that locally clusters haplotypes [19]. BEAGLE with the options applied in this study phased related individuals as if they were unrelated. Ignoring additive genetic relationships can lead to inconsistent haplotypes between related individuals. Thus, the accuracy of estimating haplotypes can be improved by using linkage information from relatives [15]. However, DAGPHASE does not reconstruct haplotypes of parents, while LINKPHASE, another algorithm from the Phasebook software package [17], could use offspring information to modify phase reconstruction in parents. Further investigation is needed to verify whether the combination of both DAGPHASE and LINKPHASE may lead to more robust results in large families.

### Assumptions for the identification of recombination events

Haldane’s (e.g., [3,10]) and Kosambi’s map functions (e.g., [2,46]) are often used to calculate recombination probabilities and to estimate the genetic length of each chromosome. In contrast, in this study crossover events were assumed to follow a binomial distribution following Karlin [30]. Haldane [47] assumed a Poisson distribution and that crossovers in adjacent intervals occur independently. Kosambi’s function [48] makes strong assumptions about interference between nearby crossovers. Kosambi’s function appears to produce more realistic map distances than Haldane’s function [29]. Both these map functions postulate that theoretically an unlimited number of crossovers can occur per chromosome. However, Karlin [30] assumed that, at most, N crossovers could be independently distributed in an interval, with the number of events following a binomial distribution. In Additional file 9: Figure S7, the autosomes were sorted by their genetic length (M), and chromosomal recombination rates produced using Karlin’s formula (with *N* = 4) were intermediate to those produced by the Kosambi and Haldane functions. Lian et al. [49] reported that crossover interference increases with decreasing chromosome length. Since strong positive crossover interference exists, quantifying the level of crossover interference on each chromosome could improve the estimation of recombination rates. For example, Broman and Weber [50] found that in human family data a gamma distribution better characterized chromosome-specific crossover interference than did four other distributions.

Many instances of double crossover events over a short distance (i.e. within 1 Mb) and individuals with excessive numbers of recombination events were observed. Genotyping, phasing, and map errors can cause overestimation of recombination rates and lead to biases in determining haplotypes from genotypic data. The data were carefully filtered for the presence of apparent double crossover events. Two crossover events separated by a small distance (<2 Mb) were attributed to phasing errors and were ignored from calculation of crossover probabilities and GRN. Other phasing or genotyping errors, such as when a sire had a recombination rate significantly higher than 0.025 in a certain window, or when all sons in a family showed two or more crossover events at the same location, were also ignored in subsequent analyses. More than two crossover events located nearby suggests a marker order error. Rather than removing the unlikely recombination event as we did here, a reordering of the markers should be considered for further study [51]. The existence of gene conversion across the chromosome is another possible cause of apparent double crossover events. During meiosis, heteroduplexes are generated in the form of either crossover or non-crossover events. Gene conversion is the non-crossover form of a heteroduplex, which is the consequence of mismatched base pairs in a heteroduplex region corrected by DNA repair mechanisms [52]. Heteroduplex regions can extend for several kb and can contain some mismatched base pairs [52]. The resolution of the SNP panel used in this study did not allow heteroduplex regions to be confirmed. Some of the double crossovers over a short distance (i.e. within 1 Mb), which we ignored may represent gene conversion. There is evidence that crossover hot spots are hot spots for gene conversion in mice [53] and humans [54].

### Impact of homozygous segments on the identification of recombination events

Each recombination event was identified within a recombination interval, which is the segment of homozygous non-informative loci that could have belonged to the haplotype of either parent. Recombination that occurs within a long homozygous segment cannot be localized. The average length of recombination intervals was 1.38 Mb (~23 SNPs) for Angus and for Limousin. The average number of SNPs in homozygous segments in sires was equal to 3.82±12.34 (~0.23±0.74 Mb) in Angus and 3.42±6.73 (~0.20±0.40 Mb) in Limousin. Long homozygous segments were defined as those containing more than 20 SNPs. On average, an autosome contained 3.4±1.3 such long segments in Angus and 1.5±0.7 in Limousin. Therefore, the impact of long homozygous segments on the identification of recombination events is not expected to be a factor in this study.

### Estimation of chromosome recombination distance per Mb and heritability of GRN

The average chromosome-specific cM per Mb ratio increased with chromosome size, as in previous studies [2,46]. Kong et al. [2] reported an average genomic recombination distance per Mb of 1.19 cM/Mb in humans. Our estimate of 1.23 cM/Mb was similar to the 1.25 cM/Mb value reported by Arias et al. [46] based on the Btau4.0 assembly [55] with a total physical length of 2468.3 Mb for the 29 autosomes, rather than 2511.4 Mb for the UMD3.1 assembly. Inconsistencies in chromosome lengths and marker order led to different chromosome genetic lengths.

The pedigree-based heritabilities in Angus (0.26) and Limousin (0.23) were slightly higher than that (0.22) reported by Sandor et al. [10] in a sample of 13 975 Dutch Holstein-Friesian bulls within three-generation paternal half-sib families. A repeatability model with GRN records for sires was considered in our study. Sandor et al. [10] fitted genome-wide recombination rates corrected for family size in an animal model. Differences in generation structures, sample sizes, and estimation models could lead to disparities in heritability estimates. Kong et al. [56] estimated a heritability of recombination rate of 0.30 in humans, which indicates that a large genetic component underlies variation in recombination rate and that the heritability of GRN differs between breeds and species.

### Genomic regions associated with GRN

GWAS have been widely applied in humans [57] and livestock [58]. Because inferences from Bayesian methods are based on the joint posterior distribution, they are useful for GWAS [59]. Regardless of the method used, detection of large-effect QTL is easier than detection of small-effect QTL. Unpublished simulations using beef cattle genotypes shows that causal mutations may lie in regions upstream or downstream of the window that has the strongest association. Although flanking regions near the most strongly associated windows (±2 Mb) were investigated, further analyses are needed to confirm our results. Significant windows associated with genome-wide recombination were located on different chromosomes in the Angus and Limousin breeds. However, Saatchi et al. [60] identified QTL of growth and production traits with consistent effects across multiple breeds. Further investigation is needed to verify whether the location and impact of recombination QTL differs between breeds. Two regions, one on BTA6 for the Angus breed and one on BTA4 for the Limousin breed, were found to explain a significant proportion of the genetic variance (>0.2%), but SNPs with the highest PPI within these two windows were not significant. However, the previous validated genes *RNF212*[10-12] and *SPO11*[4], were 4 Mb downstream from the window detected on BTA6 in Angus and 4 Mb upstream from the window detected on BTA4 in Limousin, respectively. Differences in mapping results for genome-wide recombination number (or rate) in plants [8], humans [11], dairy cattle [10], and beef cattle suggest that genome-wide recombination could be regulated in a species-specific manner, that the effects of QTL differ between species, and that the genetic determinism of regulation of recombination is probably polygenic.

### Influence of recombination on imputation accuracy and haplotype phasing

Imputation accuracy was higher in the regions with denser markers and higher LD levels (*r*^*2*^). With denser markers, better imputation accuracy is expected [24,61] and stronger LD between SNPs improves the reconstruction of haplotypes [62]. Higher recombination rates reduced the accuracy of haplotype phasing and genotype imputation and conversely, imputation accuracy was lowest in recombination hot windows.

The use of haplotypes is advantageous for genomic prediction and GWAS [16,63] provide accurately phased haplotypes. Marker location errors within a genome assembly can be detected by recombination analysis. An improved genome assembly leading to a more accurate reflection of true meiotic recombination could be produced by reordering the markers. Similarly, the accuracy of haplotype phasing and imputation from low- to high-density SNP panels could be improved by using recombination results. How to implement recombination information in haplotype phasing and imputation remains a challenging question.

## Conclusions

This study investigated the relationships between recombination, haplotype phasing, and imputation in two breeds of cattle. The accuracy of phasing using DAGPHASE was superior to BEAGLE, which did not use linkage information from parent-offspring. The major reasons for the detection of unlikely recombination events are gene conversion and phasing errors. Gene conversion is caused by mismatch correction in heteroduplex regions. Phasing errors can be influenced by limited sample size, small half-sib families, low marker density, and marker location errors in the genome assembly. The QTL mapping results for genome-wide recombination number in Angus differed from those in Limousin, which suggests that recombination is under polygenic control. High levels of recombination decrease the accuracy of phasing and genotype imputation. These results suggest that recombination analysis can detect location errors within the genome assembly, and could be used to improve the inference of haplotype phase and the accuracy of genotype imputation from low- to high-density panels.

## Competing interests

The authors declare that they have no competing interests.

## Authors’ contributions

ZW undertook the analysis and wrote the draft. MS and RDS carried out quality controls for markers. DJG conceived the study and contributed to the methods. DJG, JFT and MS contributed to the final version of manuscript. All authors read and approved the final manuscript.

## Supplementary Material

Additional file 1: Figure S1 Expected and observed crossover probabilities in Angus and Limousin. (A) Representative example of expected and observed crossover probabilities in Angus (green) and Limousin (blue) for autosome 15. (B) Probability of zero (black), one (red), two (green), three (blue), and more than three (grey) crossover events for the 29 bovine autosomes in both Angus and Limousin. Plain line represents expected probability, dashed line represents observed probability in Angus, and dotted line represents observed probability in Limousin. Click here for file

Additional file 2: Figure S2 Correlation between number of haplotypes and recombination rate within 1 Mb windows across the 29 autosomes in Angus. Click here for file

Additional file 3: Figure S3 Recombination rate within 1Mb window estimated in Angus and Limousin. (A) Representative example of the variation in recombination rate within 1 Mb windows across bovine autosome 15. The plain line (upper) corresponds to the recombination rate estimated in Angus, while the dashed line (lower) corresponds to the recombination rate estimated in Limousin. (B) Variation in recombination rate within 1 Mb windows across the 29 bovine autosomes. The plain line (upper) corresponds to the recombination rate estimated in Angus, while the dashed line (lower) corresponds to recombination rate estimated in Limousin. Click here for file

Additional file 4: Figure S4 Frequency distribution of genome-wide recombination number (GRN) in both Angus (left) and Limousin (right). Click here for file

Additional file 5: Figure S5 Distribution of genome-wide recombination number in Angus and Limousin families. (A) Representative example of distribution of genome-wide recombination number (GRN) in Angus half-sib families. Only sires with no more than 20 offspring are presented. Sires were sorted according to the number of their offspring. (B) GRN in Angus half-sib families. Black dots correspond to GRN in sons sorted by sires and red dots correspond to the average GRN for each sire. (C) GRN in Limousin half-sib families. Black dots correspond to GRN in sons sorted by sires and red dots correspond to the average GRN for each sire. Click here for file

Additional file 6: Table S1 Candidate windows and SNPs for genome-wide recombination number in Angus and Limousin. 1 Mb windows that explain a significant proportion of genetic variation (>0.2%), and results for significant SNPs and positional candidate genes within these windows detected for genome-wide recombination number in Angus and Limousin. Click here for file

Additional file 7: Table S2 Imputation accuracy, number of markers, SNP density and average LD for bovine autosomes in Angus based on UMD3.1 assembly locus coordinates. Click here for file

Additional file 8: Figure S6 Relationship between imputation accuracy and recombination rate across the 29 autosomes in Angus. (A) Average imputation accuracy in individuals with zero (red), one (green), two (blue), and more than two (purple) crossover events across the 29 autosomes. (B) Correlation between imputation accuracy and crossover rate (cM/Mb) within 1 Mb windows. Click here for file

Additional file 9: Figure S7 Chromosome-wide recombination probabilities calculated using Karlin’s (red), Haldane’s (green) and Kosambi’s (blue) map functions. Click here for file
