# Supplementary figures and images for: Recombination locations and rates in beef cattle assessed from parent-offspring pairs
Source: Genet Sel Evol. 2014 May 29;46(1):34. doi: 10.1186/1297-9686-46-34 (PMC4071795; doi:10.1186/1297-9686-46-34)

## Slide 1
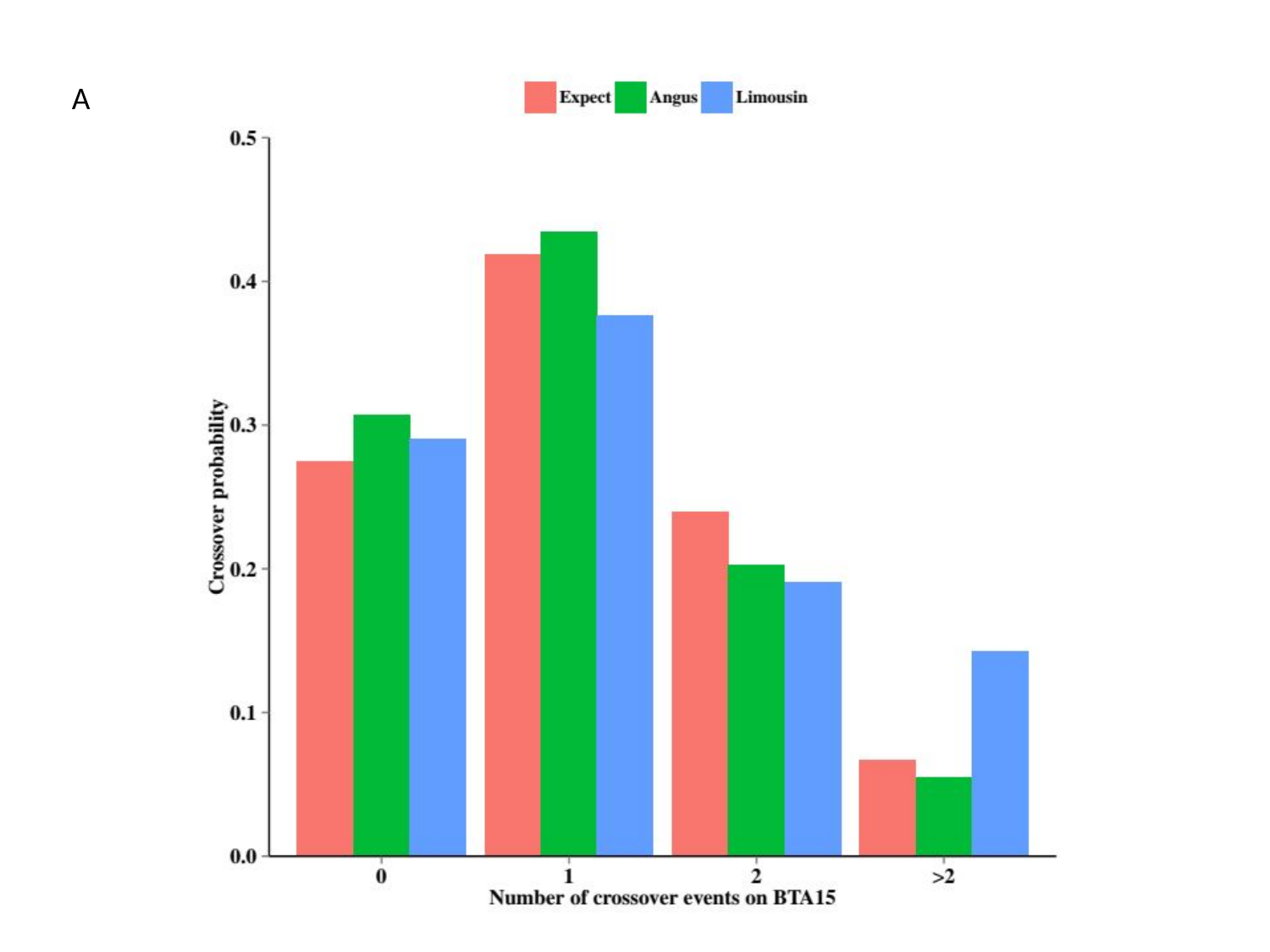

A

## Slide 2
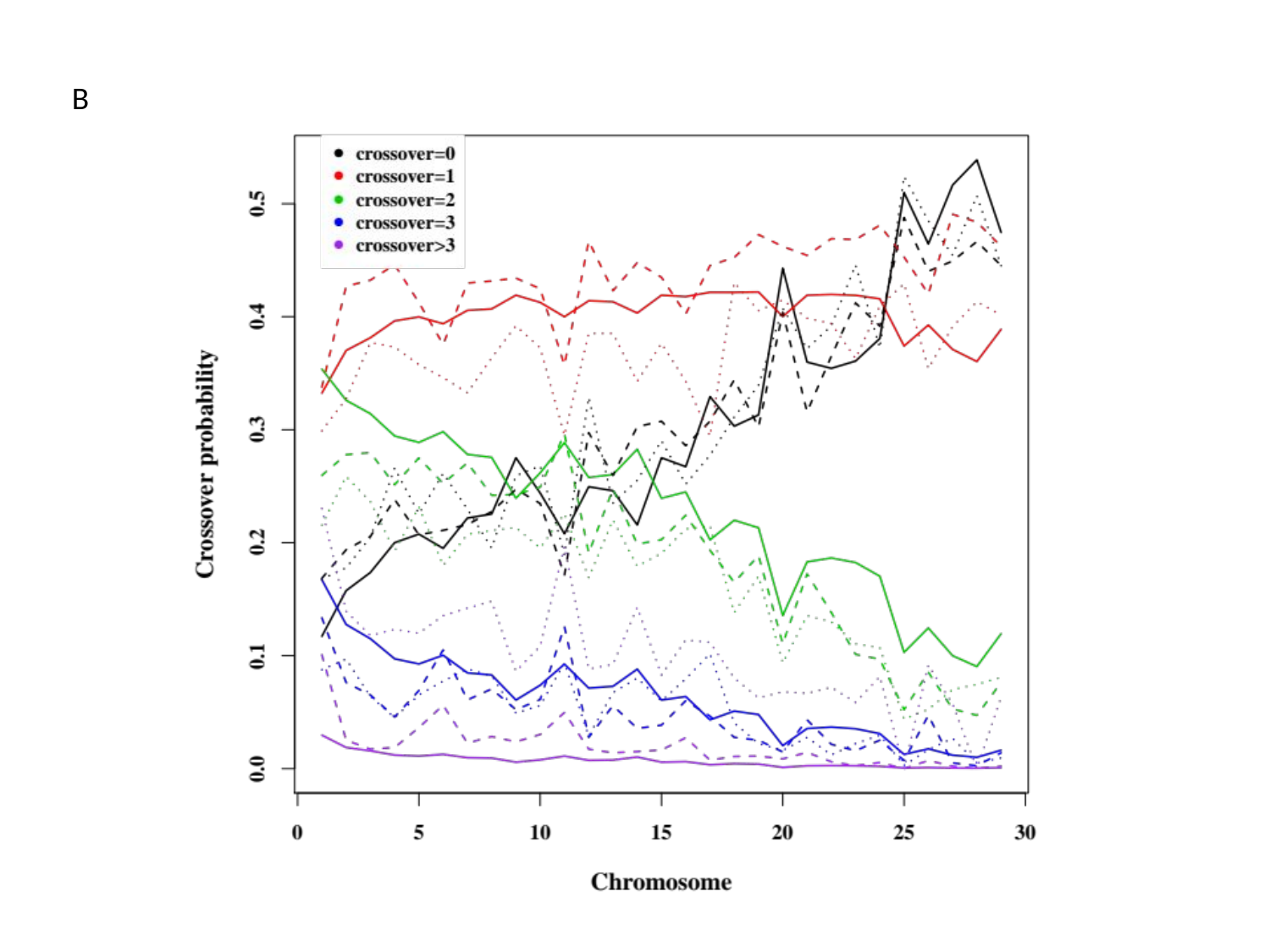

B

Supplement: Additional file 1: Figure S1 — Expected and observed crossover probabilities in Angus and Limousin. (A) Representative example of expected and observed crossover probabilities in Angus (green) and Limousin (blue) for autosome 15. (B) Probability of zero (black), one (red), two (green), three (blue), and more than three (grey) crossover events for the 29 bovine autosomes in both Angus and Limousin. Plain line represents expected probability, dashed line represents observed probability in Angus, and dotted line represents observed probability in Limousin. [file 1297-9686-46-34-S1.pptx]

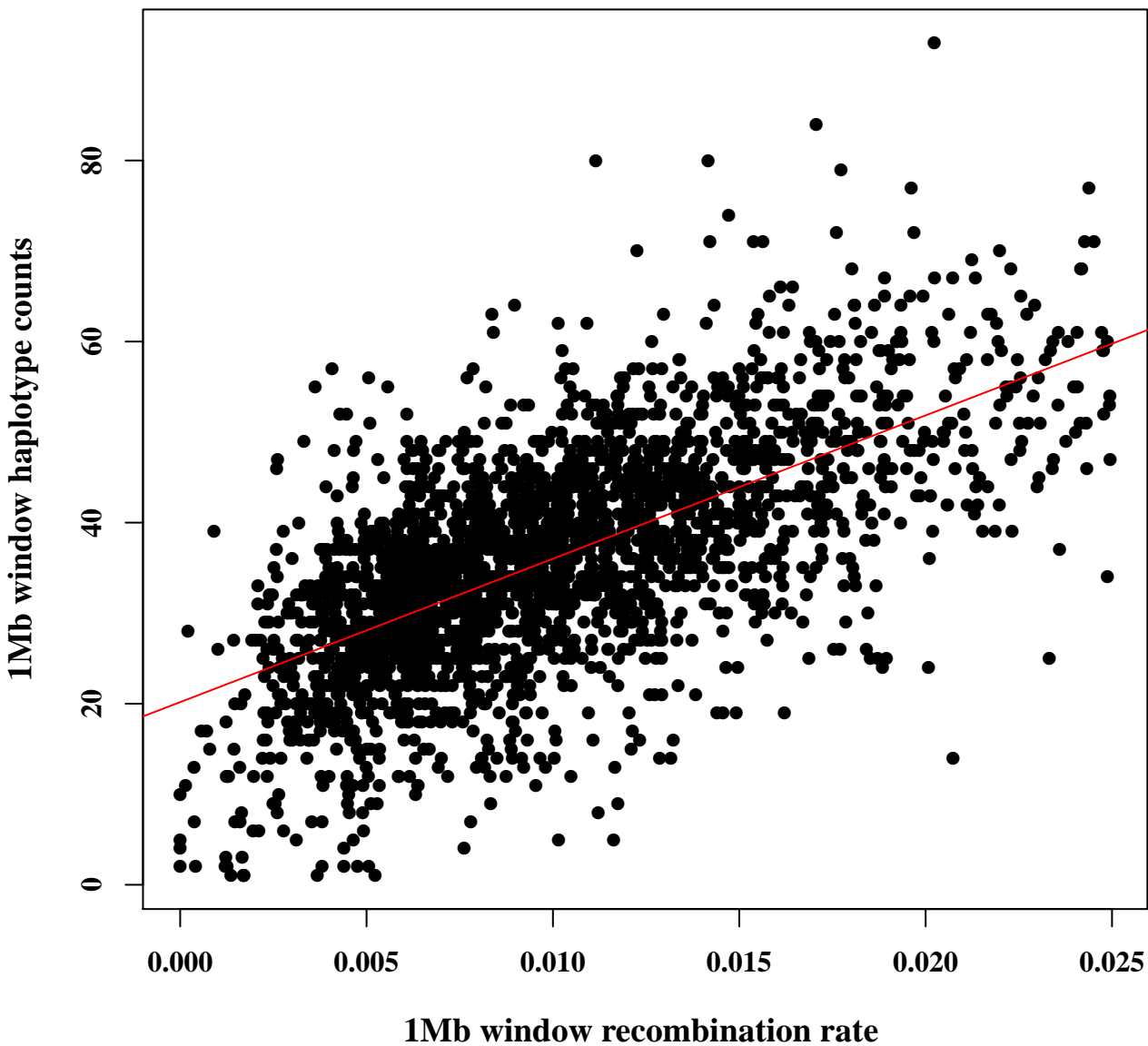

Supplement: Additional file 2: Figure S2 — Correlation between number of haplotypes and recombination rate within 1 Mb windows across the 29 autosomes in Angus. [file 1297-9686-46-34-S2.pdf]

## Slide 1
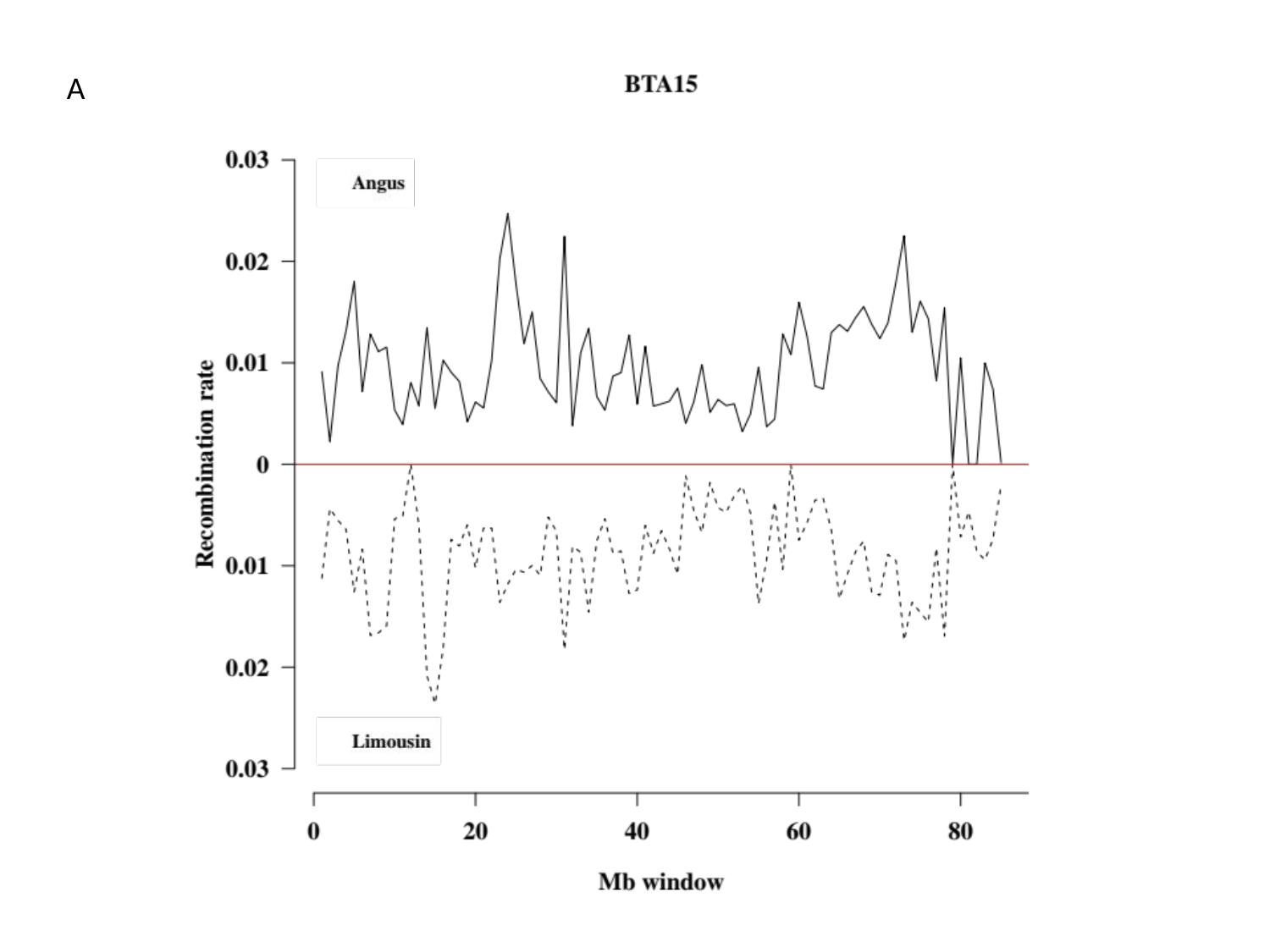

A

## Slide 2
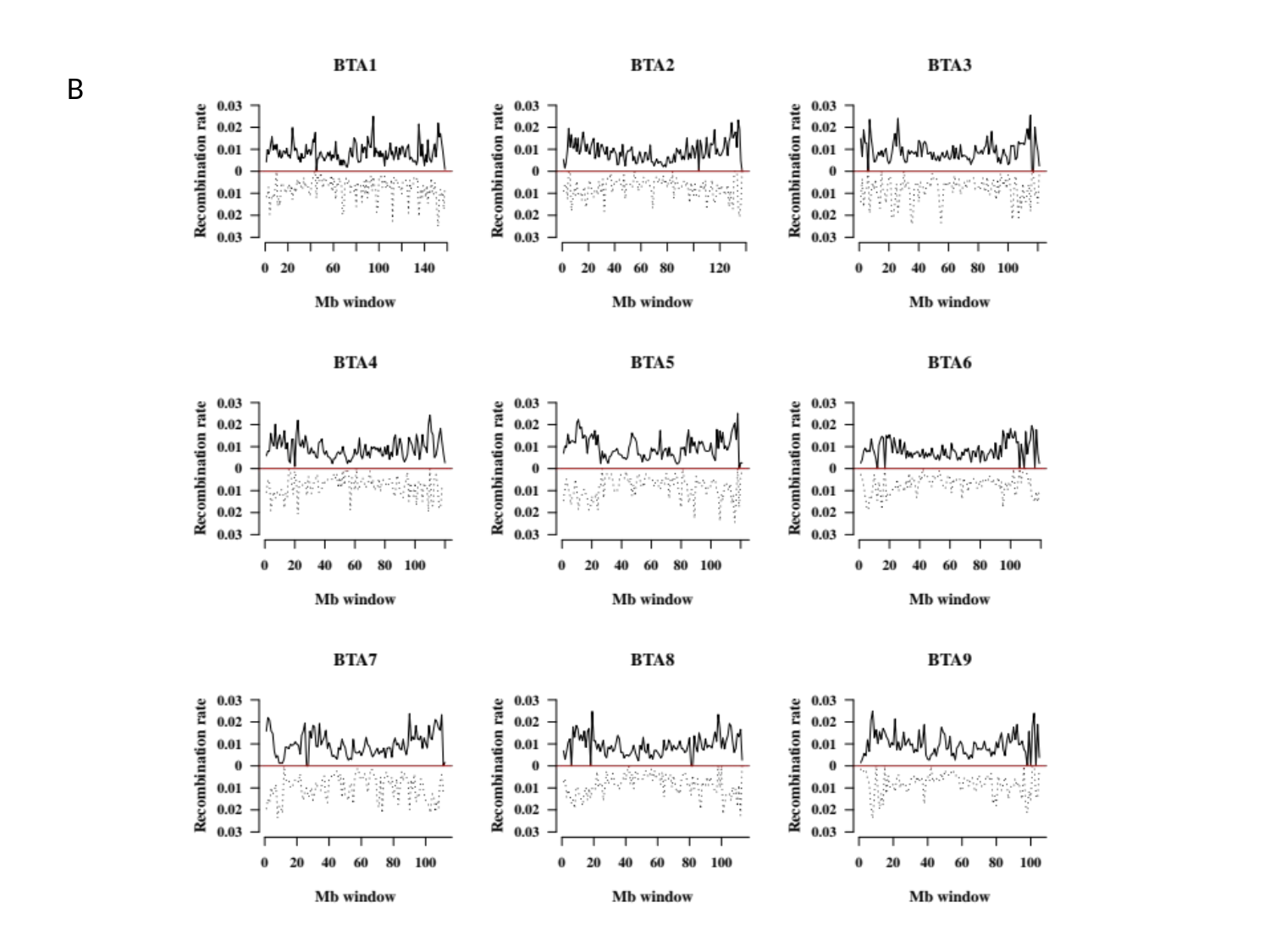

B

## Slide 3
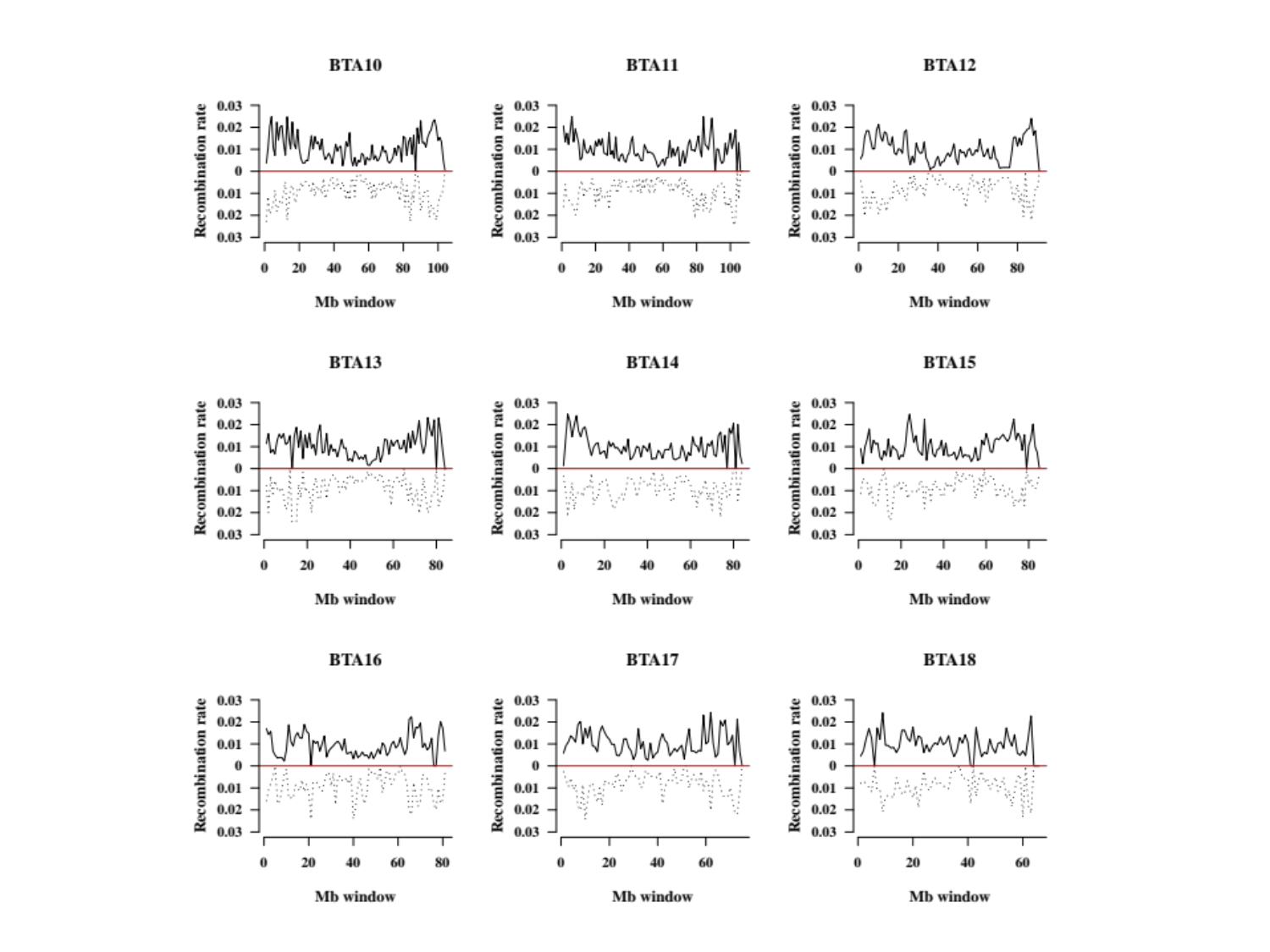

## Slide 4
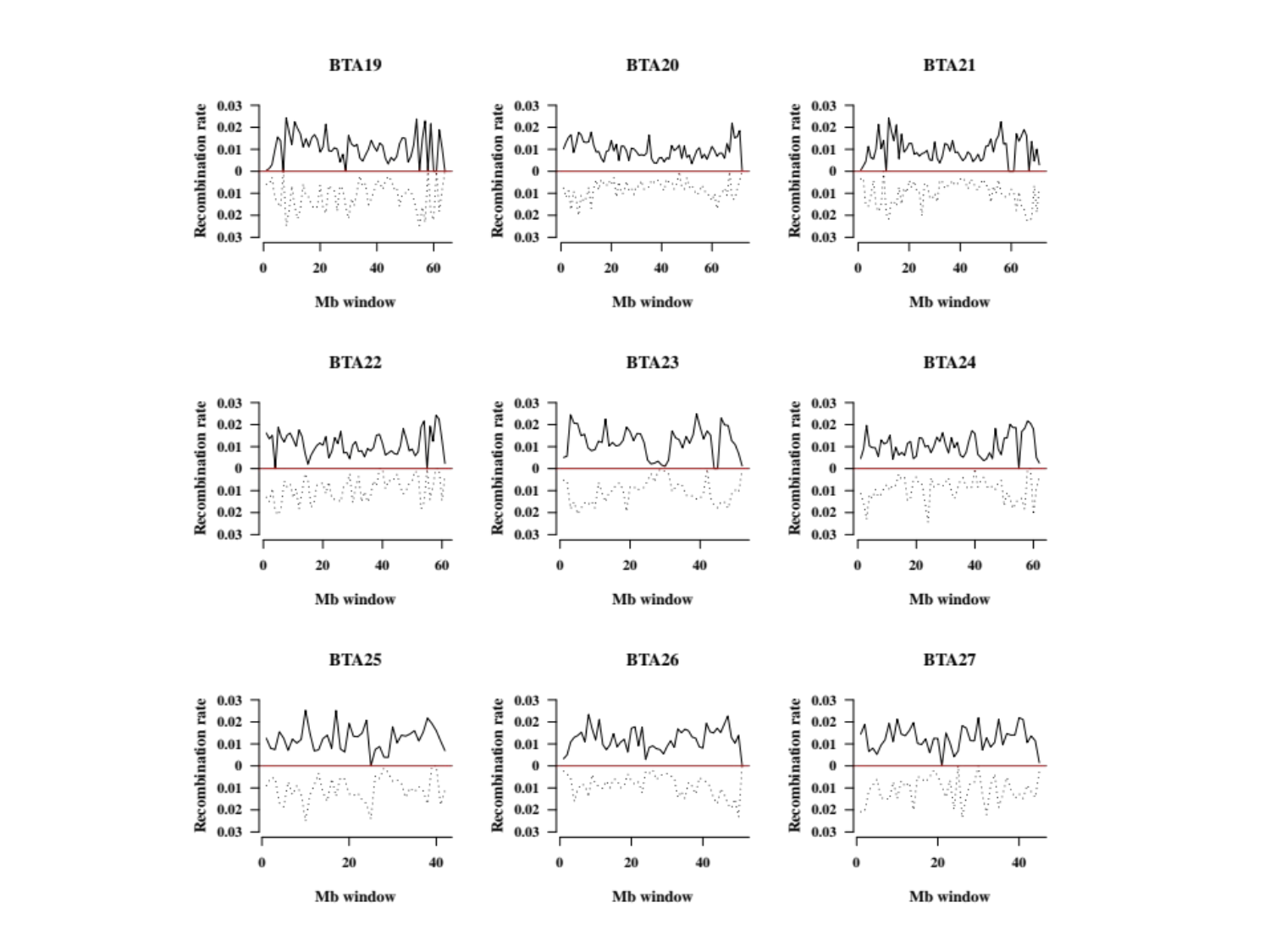

## Slide 5
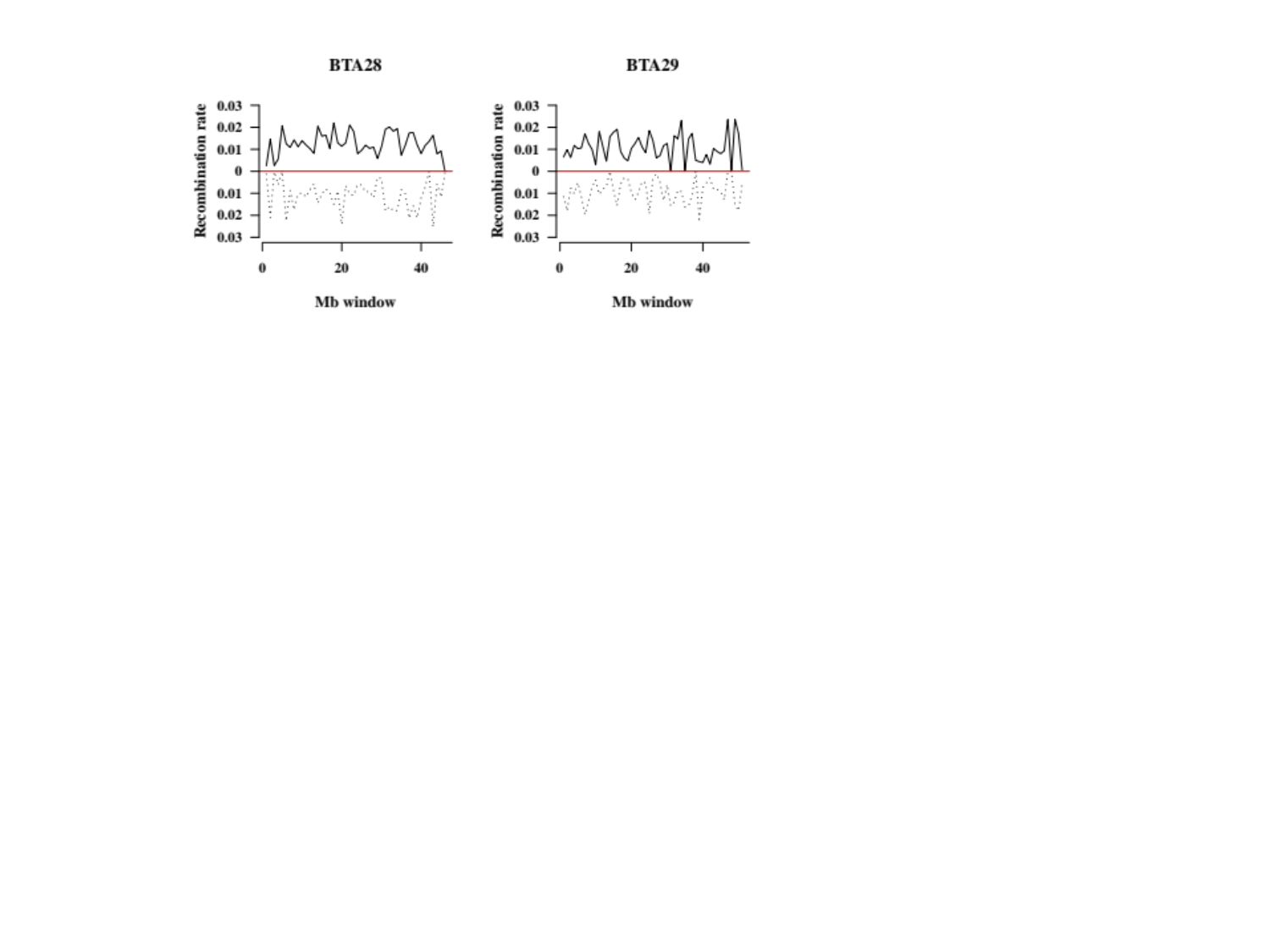

Supplement: Additional file 3: Figure S3 — Recombination rate within 1Mb window estimated in Angus and Limousin. (A) Representative example of the variation in recombination rate within 1 Mb windows across bovine autosome 15. The plain line (upper) corresponds to the recombination rate estimated in Angus, while the dashed line (lower) corresponds to the recombination rate estimated in Limousin. (B) Variation in recombination rate within 1 Mb windows across the 29 bovine autosomes. The plain line (upper) corresponds to the recombination rate estimated in Angus, while the dashed line (lower) corresponds to recombination rate estimated in Limousin. [file 1297-9686-46-34-S3.pptx]

# Genome-wide recombination number

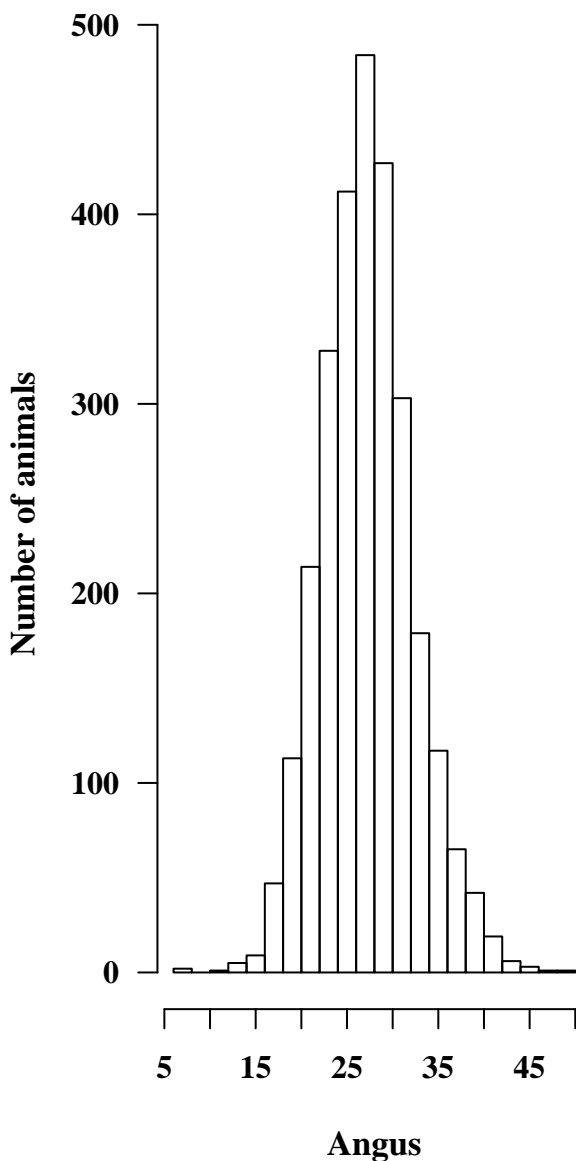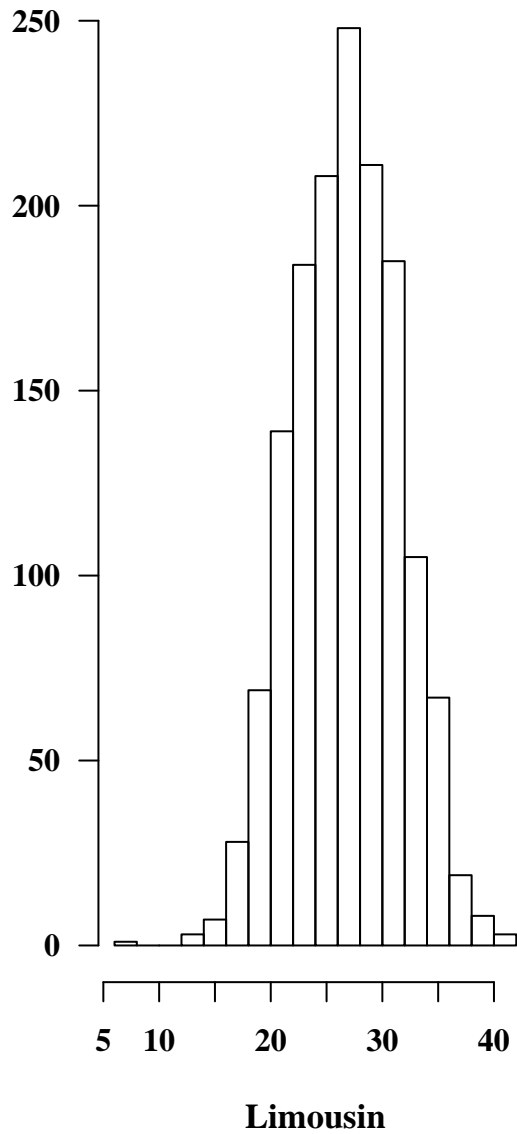

Supplement: Additional file 4: Figure S4 — Frequency distribution of genome-wide recombination number (GRN) in both Angus (left) and Limousin (right). [file 1297-9686-46-34-S4.pdf]

## Slide 1
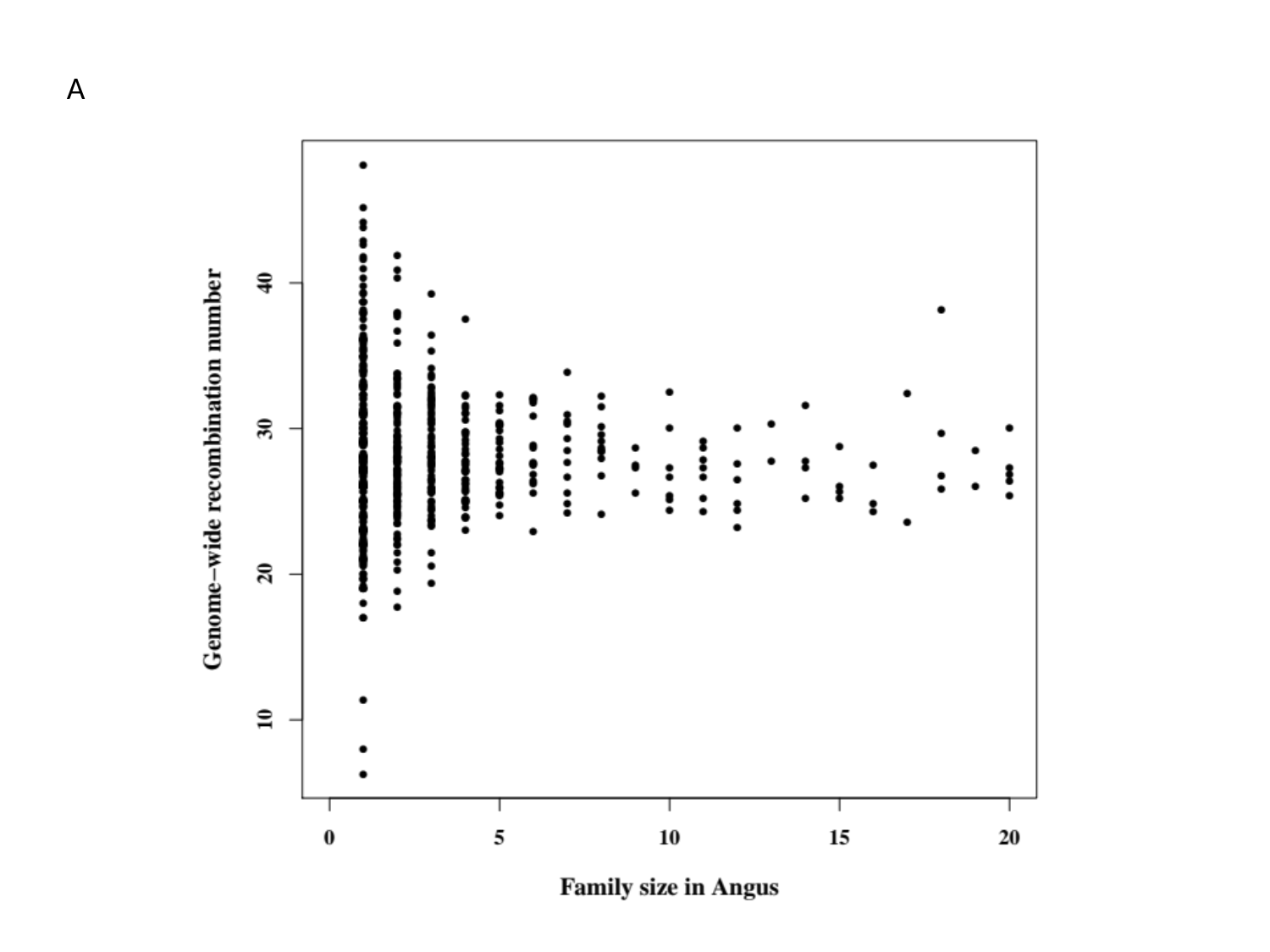

A

## Slide 2
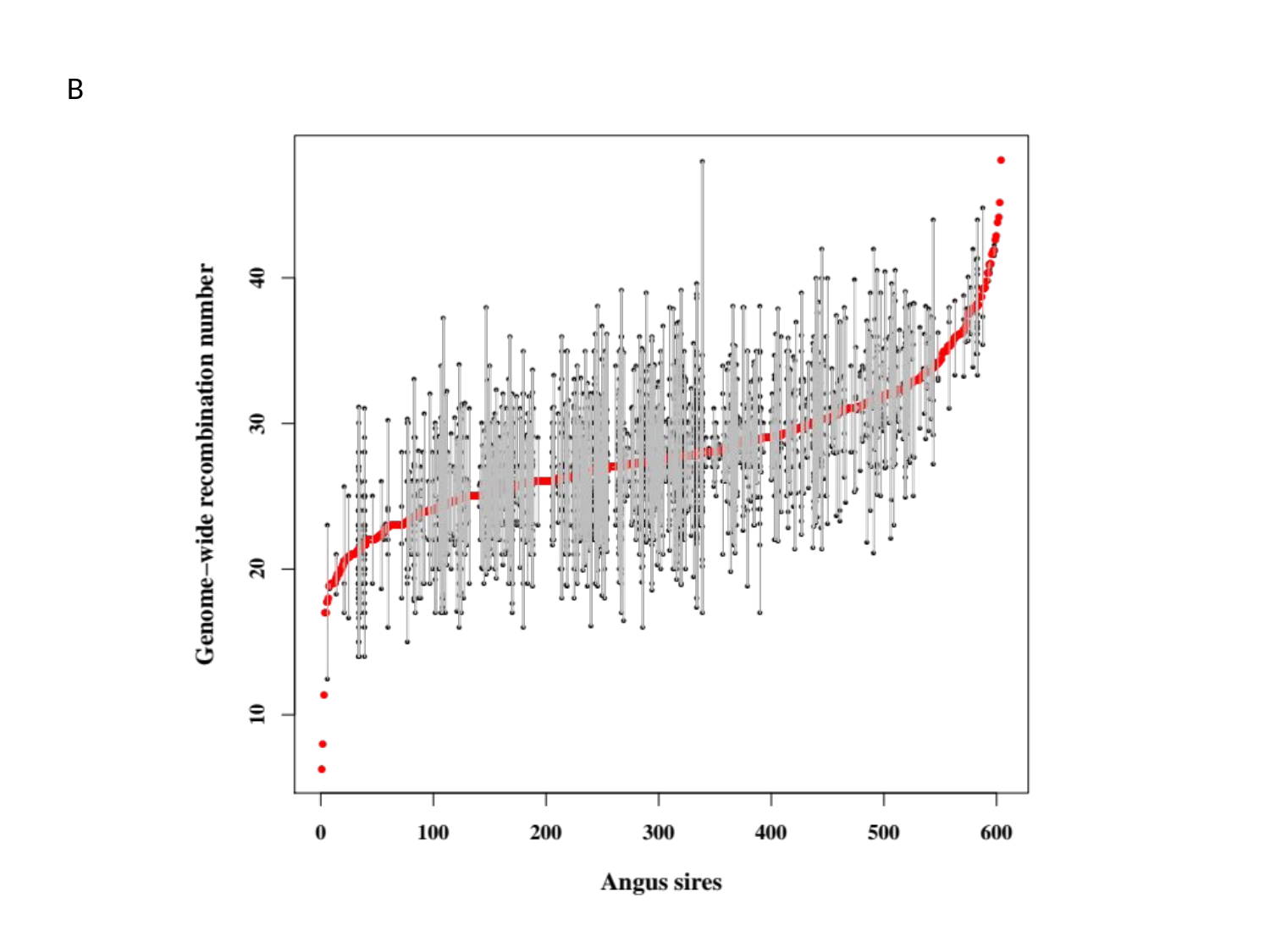

B

## Slide 3
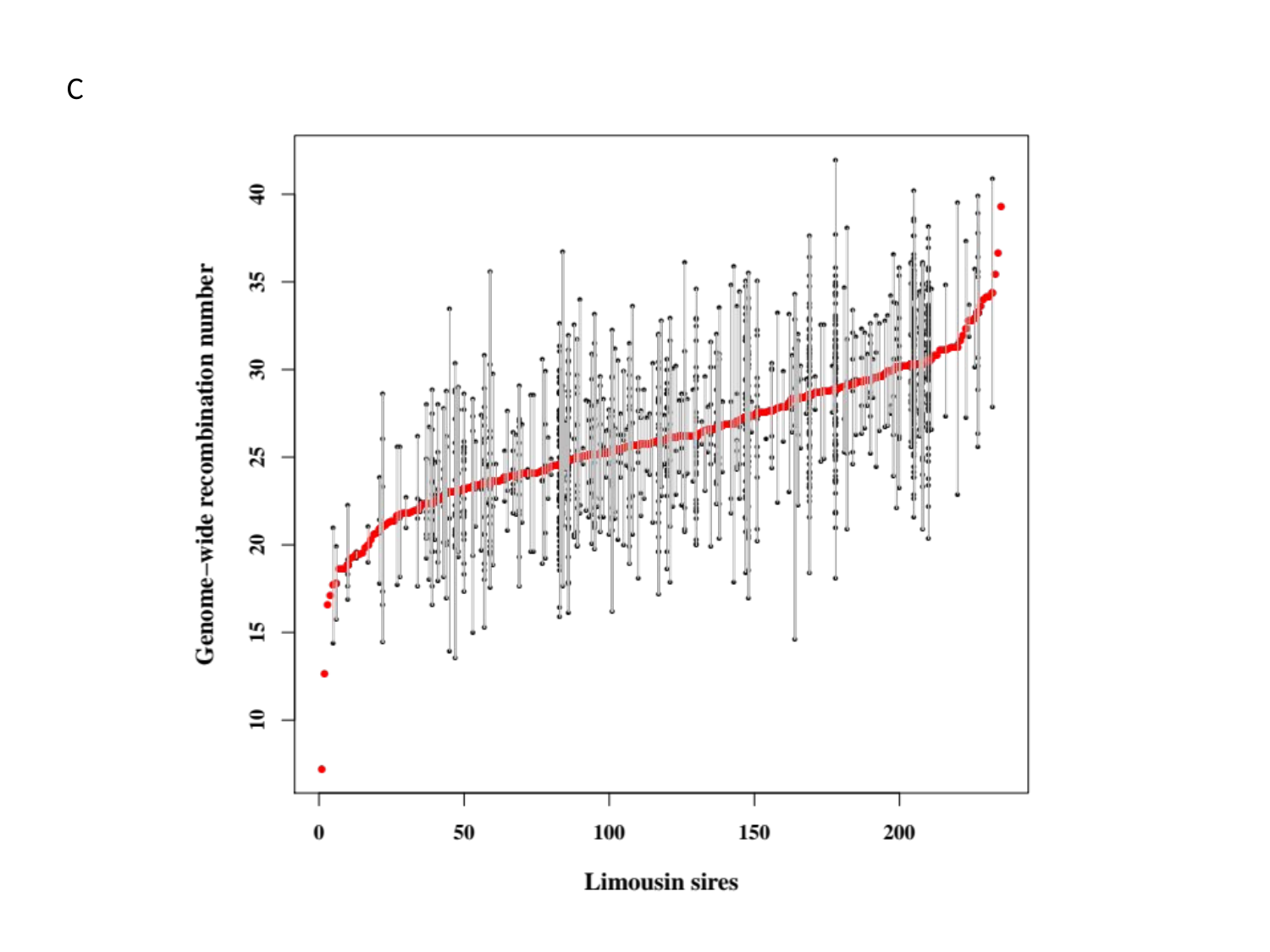

C

Supplement: Additional file 5: Figure S5 — Distribution of genome-wide recombination number in Angus and Limousin families. (A) Representative example of distribution of genome-wide recombination number (GRN) in Angus half-sib families. Only sires with no more than 20 offspring are presented. Sires were sorted according to the number of their offspring. (B) GRN in Angus half-sib families. Black dots correspond to GRN in sons sorted by sires and red dots correspond to the average GRN for each sire. (C) GRN in Limousin half-sib families. Black dots correspond to GRN in sons sorted by sires and red dots correspond to the average GRN for each sire. [file 1297-9686-46-34-S5.pptx]

## Slide 1
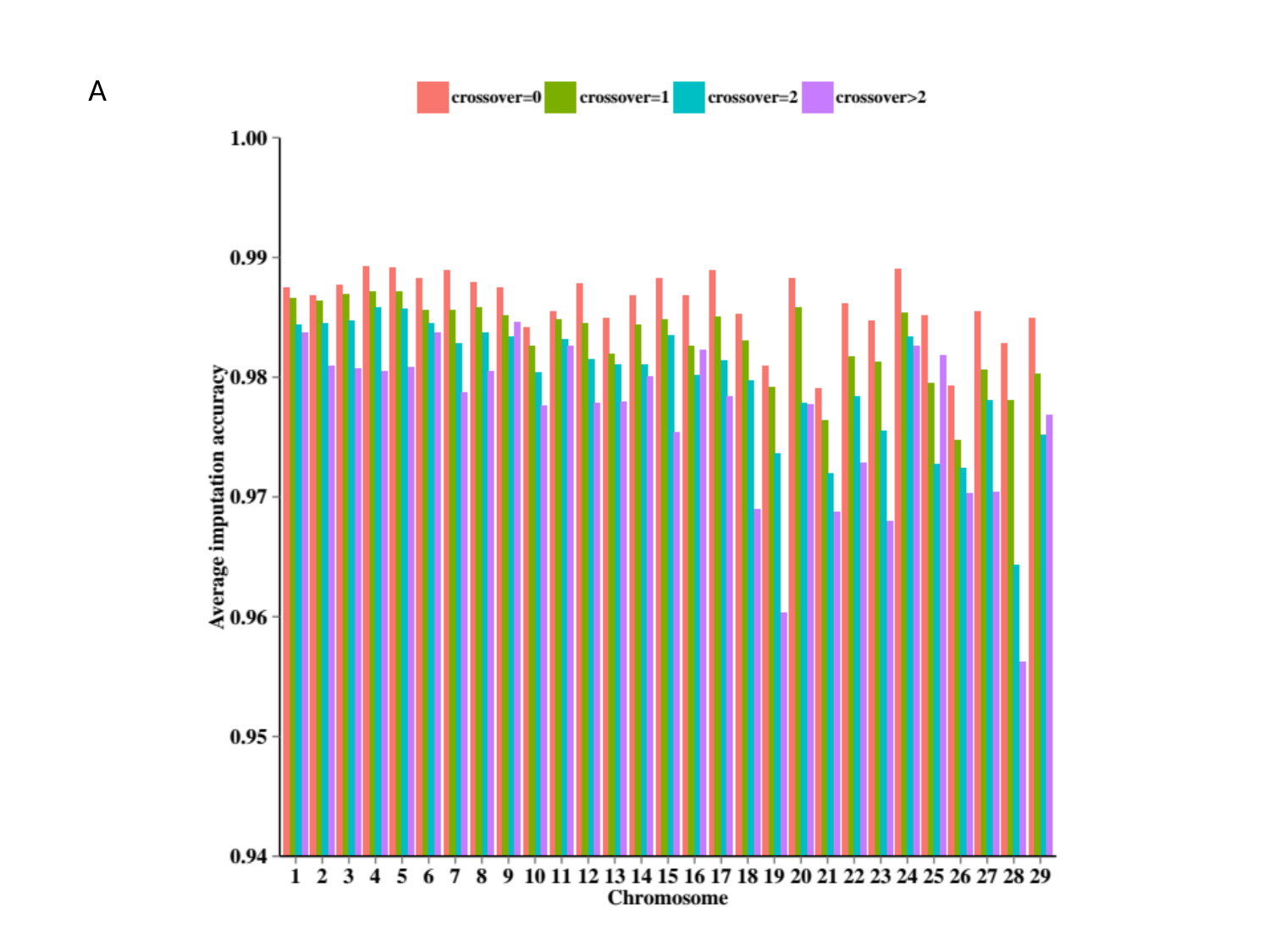

A

## Slide 2
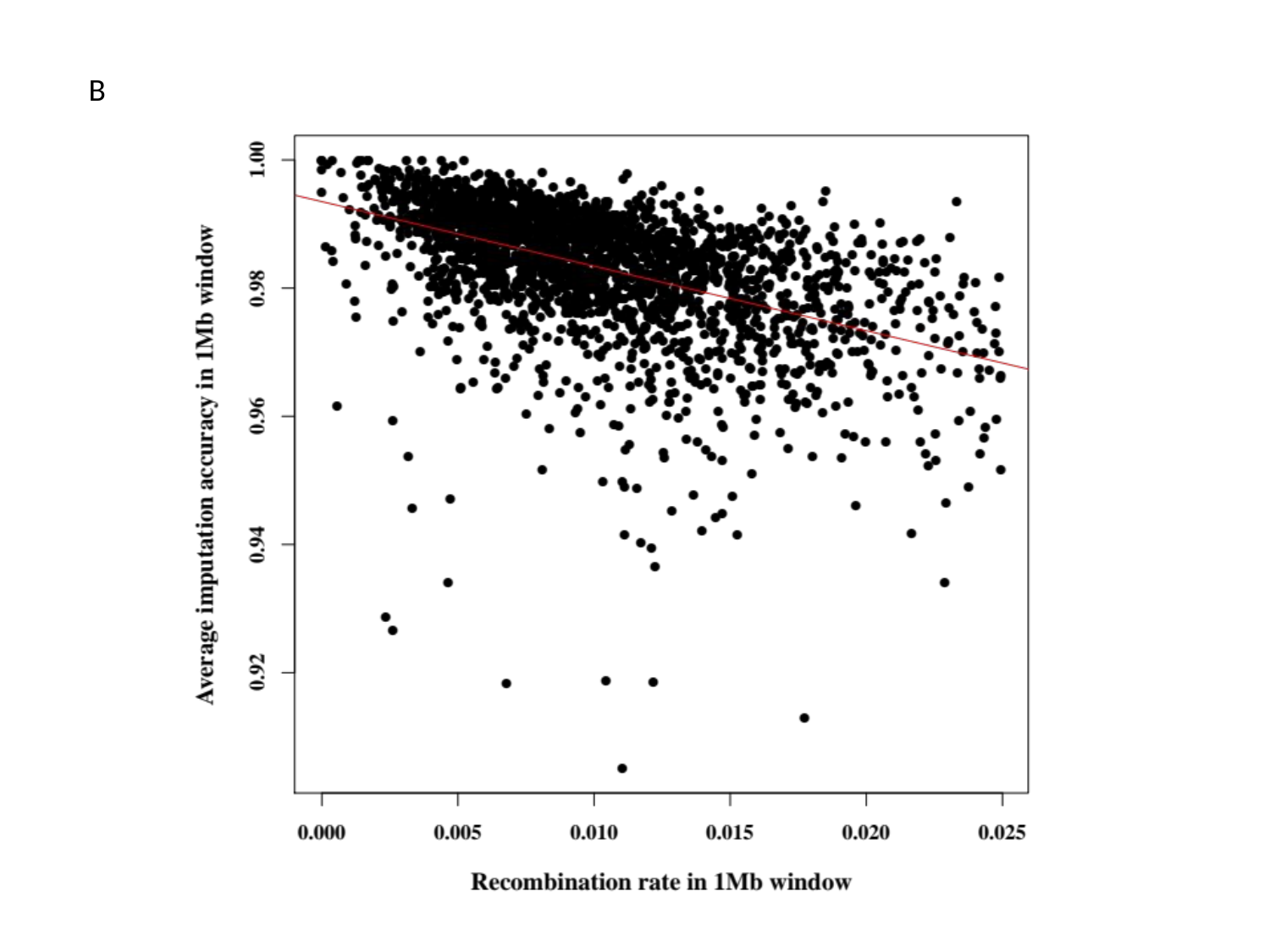

B

Supplement: Additional file 8: Figure S6 — Relationship between imputation accuracy and recombination rate across the 29 autosomes in Angus. (A) Average imputation accuracy in individuals with zero (red), one (green), two (blue), and more than two (purple) crossover events across the 29 autosomes. (B) Correlation between imputation accuracy and crossover rate (cM/Mb) within 1 Mb windows. [file 1297-9686-46-34-S8.pptx]

Probability of Recombination

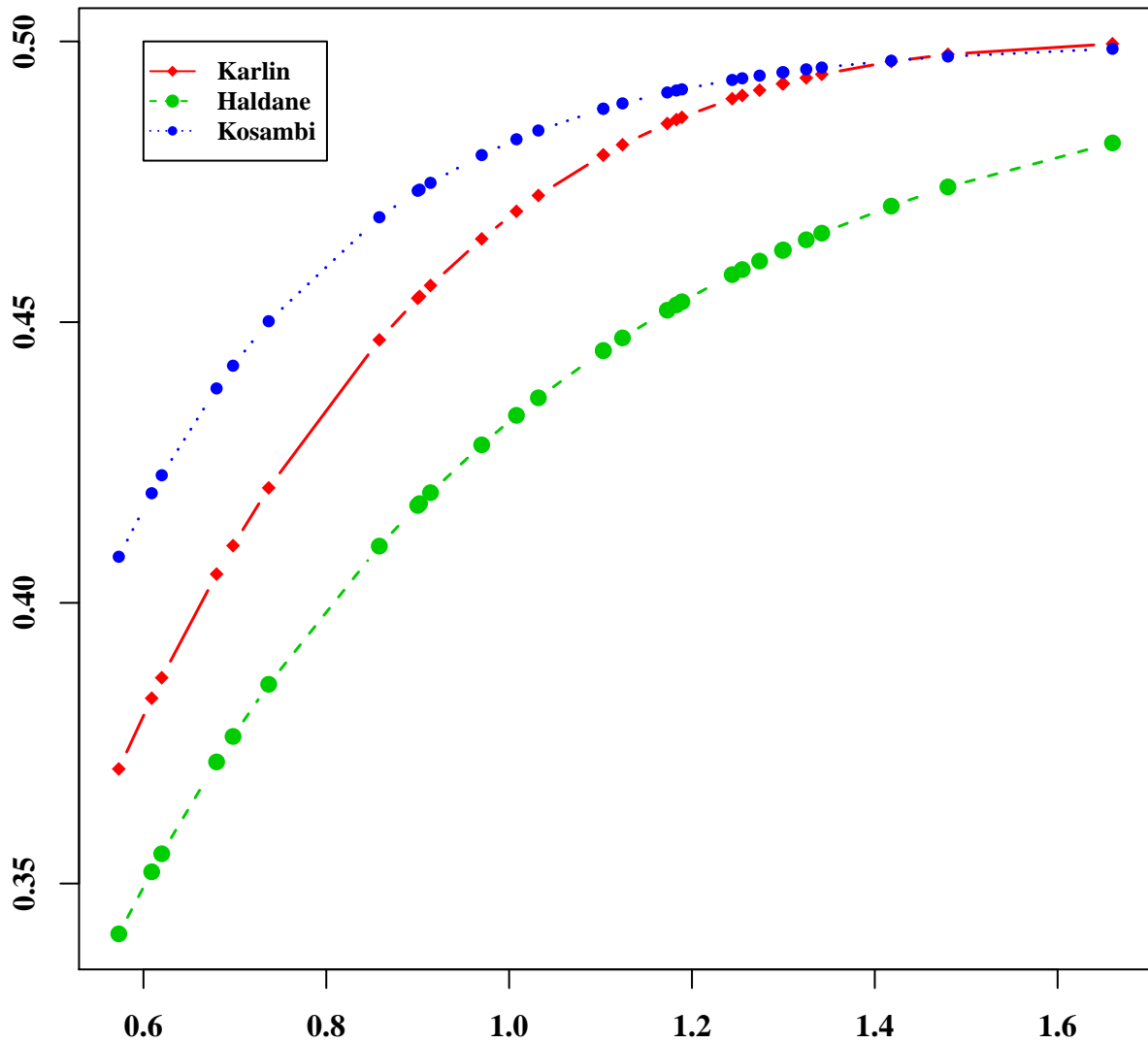

Genetic length of Chromosome (Morgan)

Supplement: Additional file 9: Figure S7 — Chromosome-wide recombination probabilities calculated using Karlin’s (red), Haldane’s (green) and Kosambi’s (blue) map functions. [file 1297-9686-46-34-S9.pdf]
