# Supplementary material for: Recombination locations and rates in beef cattle assessed from parent-offspring pairs
Source: Genet Sel Evol. 2014 May 29;46(1):34. doi: 10.1186/1297-9686-46-34 (PMC4071795; doi:10.1186/1297-9686-46-34)
Supplement: Additional file 6: Table S1 — Candidate windows and SNPs for genome-wide recombination number in Angus and Limousin. 1 Mb windows that explain a significant proportion of genetic variation (>0.2%), and results for significant SNPs and positional candidate genes within these windows detected for genome-wide recombination number in Angus and Limousin. [file 1297-9686-46-34-S6.docx]

**Table S1** Candidate windows and SNPs for genome-wide recombination number in Angus and Limousin.

1 Mb windows that explain a significant proportion of genetic variation (> 0.2%), and results for significant SNPs and positional candidate genes within these windows detected for genome-wide recombination number in Angus and Limousin.

|  | Significant 1 Mb window | | | | | Significant SNP | | | | | Candidate Gene | |
| --- | --- | --- | --- | --- | --- | --- | --- | --- | --- | --- | --- | --- |
| Breed | BTA | Window (Mb) | % genetic variance | Number of SNPs | WPPA | SNP | Position  (kb) | PPI | % genetic variance | *p*-value in model | Gene | Distance (Mb) |
| Angus | 5 | 106 | 0.27 | 18 | 0.14 | ARS-BFGL-NGS-54496 | 106178425 | 0.11 | 0.08 | 0.01 | *PRMT8*  *RAD52C* | +0.6  +2.0 |
|  | 6 | 115 | 0.24 | 26 | 0.19 | Hapmap39435-BTA-77865 | 115229156 | 0.11 | 0.20 | 0.11 | *RNF212* | -4.0 |
|  | 19 | 10 | 0.33 | 14 | 0.13 | UA-IFASA-6593 | 10920596 | 0.18 | 0.23 | 1.4*10^-4^ | *RAD51C* | -1.0 |
|  | 20 | 12-13 | 0.80 | 38 | 0.36 | ARS-BFGL-NGS-60738 | 12752857 | 0.18 | 0.29 | 0.037 | *RAD17* | -1.7 |
|  | 21 | 67 | 3.48 | 22 | 0.45 | ARS-BFGL-NGS-105314 | 67195140 | 0.45 | 3.42 | 0.0077 | *XRCC3* | +2.0 |
|  | 22 | 3 | 2.63 | 18 | 0.51 | ARS-BFGL-NGS-30499 | 3046992 | 0.28 | 1.09 | 0.031 |  |  |
|  | 24 | 39 | 1.71 | 18 | 0.36 | ARS-BFGL-NGS-4629 | 39876972 | 0.33 | 1.24 | 0.0057 | *PTPRM* | +0.8 |
| Limousin | 4 | 89 | 2.55 | 17 | 0.25 | BTB-01759027 | 89850655 | 0.26 | 2.44 | 0.034 |  |  |
|  | 13 | 55 | 0.20 | 16 | 0.11 | ARS-BFGL-NGS-4016 | 55808723 | 0.10 | 0.15 | 0.70 | SPO11 | +4.0 |

WPPA, window posterior probability of association; PPI, posterior probability of inclusion of the selected SNP; p-value adjusted by Bonfferoni test, significance of the selected SNPs in single SNP analysis; distance, approximate distance (Mb) between the positions of the candidate gene and significant SNP.
