# Supplementary material for: Recombination locations and rates in beef cattle assessed from parent-offspring pairs
Source: Genet Sel Evol. 2014 May 29;46(1):34. doi: 10.1186/1297-9686-46-34 (PMC4071795; doi:10.1186/1297-9686-46-34)
Supplement: Additional file 7: Table S2 — Imputation accuracy, number of markers, SNP density and average LD for bovine autosomes in Angus based on UMD3.1 assembly locus coordinates. [file 1297-9686-46-34-S7.docx]

**Table S2** Imputation accuracy, number of markers, SNP density and average LD for bovine autosomes in Angus based on UMD3.1 assembly locus coordinates.

| Chromosome | Imputation Accuracy | # of markers | Marker density (kb) | $r^{2}$ in 1Mb window |
| --- | --- | --- | --- | --- |
| 1 | 0.983 ± 0.007 | 2603 | 60.83 | 0.260 ± 0.303 |
| 2 | 0.984 ± 0.007 | 2221 | 61.71 | 0.234 ± 0.290 |
| 3 | 0.984 ± 0.008 | 2026 | 59.94 | 0.259 ± 0.305 |
| 4 | 0.985 ± 0.006 | 1984 | 60.58 | 0.248 ± 0.295 |
| 5 | 0.985 ± 0.007 | 1710 | 70.87 | 0.257 ± 0.307 |
| 6 | 0.984 ± 0.008 | 2027 | 58.93 | 0.258 ± 0.306 |
| 7 | 0.983 ± 0.007 | 1750 | 64.36 | 0.269 ± 0.311 |
| 8 | 0.983 ± 0.009 | 1803 | 62.89 | 0.254 ± 0.300 |
| 9 | 0.983 ± 0.007 | 1650 | 64.07 | 0.232 ± 0.293 |
| 10 | 0.980 ± 0.008 | 1731 | 60.26 | 0.217 ± 0.284 |
| 11 | 0.982 ± 0.009 | 1830 | 58.64 | 0.269 ± 0.305 |
| 12 | 0.983 ± 0.008 | 1344 | 67.83 | 0.234 ± 0.284 |
| 13 | 0.978 ± 0.011 | 1440 | 58.50 | 0.261 ± 0.303 |
| 14 | 0.983 ± 0.008 | 1455 | 58.18 | 0.268 ± 0.310 |
| 15 | 0.984 ± 0.008 | 1304 | 65.41 | 0.226 ± 0.287 |
| 16 | 0.981 ± 0.009 | 1352 | 60.45 | 0.236 ± 0.296 |
| 17 | 0.983 ± 0.008 | 1273 | 59.04 | 0.216 ± 0.272 |
| 18 | 0.980 ± 0.009 | 1061 | 62.21 | 0.251 ± 0.294 |
| 19 | 0.975 ± 0.011 | 1081 | 59.26 | 0.231 ± 0.277 |
| 20 | 0.984 ± 0.008 | 1223 | 58.91 | 0.232 ± 0.285 |
| 21 | 0.973 ± 0.010 | 1115 | 64.21 | 0.233 ± 0.286 |
| 22 | 0.980 ± 0.008 | 1041 | 59.02 | 0.236 ± 0.290 |
| 23 | 0.980 ± 0.008 | 857 | 61.30 | 0.197 ± 0.254 |
| 24 | 0.984 ± 0.007 | 1036 | 60.54 | 0.251 ± 0.300 |
| 25 | 0.980 ± 0.009 | 786 | 54.59 | 0.214 ± 0.267 |
| 26 | 0.975 ± 0.010 | 879 | 58.80 | 0.211 ± 0.279 |
| 27 | 0.981 ± 0.009 | 769 | 59.05 | 0.232 ± 0.285 |
| 28 | 0.976 ± 0.011 | 773 | 59.91 | 0.199 ± 0.246 |
| 29 | 0.979 ± 0.009 | 866 | 59.47 | 0.192 ± 0.243 |
